# Flow‐Induced Long‐Term Stable Slippery Surfaces

**DOI:** 10.1002/advs.201900019

**Published:** 2019-04-07

**Authors:** Philipp Baumli, Hannu Teisala, Hoimar Bauer, Diana Garcia‐Gonzalez, Viraj Damle, Florian Geyer, Maria D'Acunzi, Anke Kaltbeitzel, Hans‐Jürgen Butt, Doris Vollmer

**Affiliations:** ^1^ Max Planck Institute for Polymer Research Ackermannweg 10 55128 Mainz Germany; ^2^ Physics of Fluids Group University of Twente Drienerlolaan 5 7522NB Enschede The Netherlands; ^3^ School for Engineering of Matter, Transport and Energy Arizona State University Tempe AZ 85287‐1604 USA

**Keywords:** confocal microscopy, emulsions, flow, porous surfaces, wetting

## Abstract

Slippery lubricant‐infused surfaces allow easy removal of liquid droplets on surfaces. They consist of textured or porous substrates infiltrated with a chemically compatible lubricant. Capillary forces help to keep the lubricant in place. Slippery surfaces hold promising prospects in applications including drag reduction in pipes or food packages, anticorrosion, anti‐biofouling, or anti‐icing. However, a critical drawback is that shear forces induced by flow lead to depletion of the lubricant. In this work, a way to overcome the shear‐induced lubricant depletion by replenishing the lubricant from the flow of emulsions is presented. The addition of small amounts of positively charged surfactant reduces the charge repulsion between the negatively charged oil droplets contained in the emulsion. Attachment and coalescence of oil droplets from the oil‐in‐water emulsion at the substrate surface fills the structure with the lubricant. Flow‐induced lubrication of textured surfaces can be generalized to a broad range of lubricant–solid combinations using minimal amounts of oil.

From everyday experience, we know that friction is significantly reduced when driving on a wet street or moving over a slippery plank. The reason is that the rough surface is infiltrated with a liquid, also termed lubricant. For slippery lubricant‐infused porous surfaces (SLIPS),^1^, ^2^ an immobilized lubricant surface is established through capillary forces, which keep the lubricant in place within the texture.^3^, ^4^ As a consequence, a smooth lubricant surface is formed. Slippery lubricant‐infused surfaces, however, are by no means fully explored. Complexity arises from the 4‐component nature of the system (solid substrate, lubricant, liquid to be repelled, and air).

A significant challenge for applications^5^ of lubricant‐impregnated slippery surfaces is the depletion of lubricant.^6^, ^7^, ^8^, ^9^ Drag reduction is no longer in effect on lubricant‐depleted surfaces. Depletion of lubricant due to gravitational drainage, evaporation, or cloaking can be prevented or minimized by well‐thought choice of lubricant and surface design. However, no solution against shear‐induced depletion of lubricant has been presented, yet.^3^, ^8^ Hydrodynamics necessitate that a moving liquid takes lubricant along due to the requirement of continuity of the interfacial velocities and shear stresses across the liquid–lubricant interface. This gives rise to depletion of lubricant and loss of the surface functionality. Therefore, being able to replenish the lubricant is vital; to which besides spraying, dipping, or painting no strategy exists yet. These strategies require ex situ accessibility. Here, we introduce the first in situ approach of replenishing lubricant utilizing flow of oil‐in‐water emulsions.

Emulsion‐ and surfactant‐based products are ubiquitous. Emulsions constitute a large proportion of liquids that we interact with. Examples include emulsified food such as milk and yogurt, cosmetic products such as creams and lotions, or crude oils that are often delivered as water‐in‐oil emulsions.

We demonstrate the formation of a slippery surface via the flow of an emulsion over a fully water‐filled micropillar array, i.e., starting replenishing lubricant from the worst‐case scenario of a completely lubricant‐depleted surface. Intuitively, replenishing lubricant with emulsions does not seem to be possible because of hydrodynamics (no‐slip boundary conditions); a stable water film surrounds the pillars, preventing coalescence of the oil drops with the surface. In addition, buoyancy drives many lubricants away from the solid. We overcome these issues by manipulating the wettability properties of the oil drops. Laser scanning confocal microscopy (LSCM) reveals that above a threshold velocity the oil droplets attach to the pillar walls, grow larger, and finally coalesce and descend to fill the structure.

We conducted all experiments using rectangular flow cells having plastic side and top walls, **Figure** **1** (and Figure S1, Supporting Information). If not stated otherwise, the glass bottom of the flow cells contains uniform and regular SU‐8 photoresist micropillar arrays (**Figure** **2**j,k, the Experimental Section for details) hydrophobized by chemical vapor deposition (CVD) of trichlorooctylsilane (OTS, C_8_H_17_SiCl_3_). The flow cells have a height of *H* = 500 µm, a length of *L* = 17 mm, and a width of *W* = 3.8 mm, thus they are much deeper than the 10–25 µm high pillars. Hence, the flow profile is approximately parabolic through its depth. The maximal volumetric flow rate *Q* established through a flow channel using a peristaltic pump amounted to *Q* = 7.0 ± 0.2 mL min^−1^, which resulted in average flow velocity of 77 ± 2 mm s^−1^ (Figures S1 and S2, and Table S1, Supporting Information). We investigated the attachment and growth of drops to the pillar walls using an inverted laser scanning confocal microscope (Leica TCS SP8 SMD). Imaging was performed in the middle of the horizontal flow cell with respect to the lateral direction parallel to the flow.

**Figure 1:**
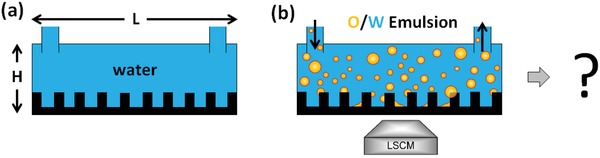
Experimental setup. a) An initially water‐filled flow channel containing a micropillar array, b) is subjected to a continuous flow of an oil‐in‐water (O/W) emulsion. Can the oil gradually replace the water in‐between the micropillars leading to the formation of a slippery surface? The filling process is monitored using an inverted laser scanning confocal microscope (LSCM).

**Figure 2:**
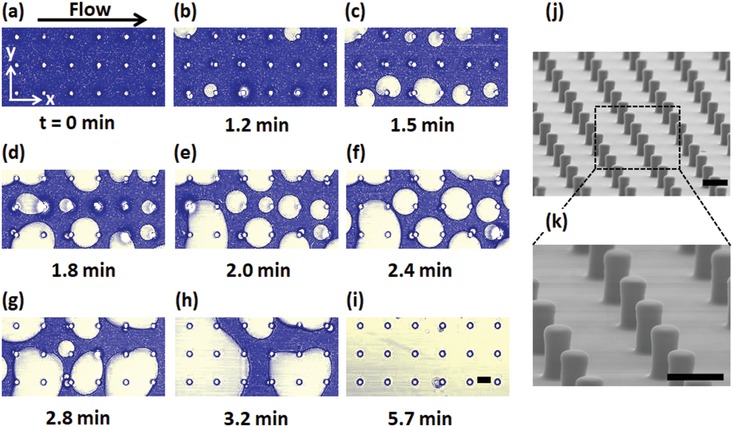
Time evolution of top view laser scanning confocal images (40×/1.11 water immersion objective lens) demonstrating complete filling (flow direction from left to right) of a micropillar array. The time series consists of an overlay of the transmission and fluorescence channel images which were simultaneously recorded. The confocal microscope imaging was focused roughly 5 µm above the bottom of the micropillar structure. An oil‐in‐water emulsion (2 wt.% of silicone oil, viscosity: 50 cSt, density: 0.96 g mL^−1^) is circulated over the micropillar array at an average flow velocity of 77 ± 2 mm s^−1^. 500 µg L^−1^ CTAB or ≈0.14% of critical micelle concentration (CMC) was added to the water phase before emulsification. a) Starting from an initially water‐filled (blue) channel, b–i) the continuous flow of emulsion leads to the attachment of oil droplets (yellow) to the pillars and the bottom substrate leading to gradual filling of the structure with oil. The water is dyed with 1 µg g^−1^ Atto 488 NHS‐Ester. The dye concentration is sufficiently low, not to change the interfacial tension. The pillars (grey) are added based on their position and size given by the transmission image. j,k) SEM images of micropillar arrays. Pillar dimensions: diameter *d* = 5 µm, center‐to‐center spacing *P* = 20 µm, and pillar height *h* = 10 µm. All scale bars are 10 µm.

In the beginning, the micropillar array is completely filled with water (blue), representing the worst‐case scenario of a slippery surface completely devoid of lubricant (Figures 1a and 2a). Subsequently, an oil‐in‐water emulsion is circulated over the micropillar array (Figure 1b). The emulsification was done using a tip sonicator (SONIFIER W‐450D) for 2 min (see the Experimental Section and Figure S3a, Supporting Information, for details). The diameter of the polydisperse oil droplets was determined by laser scanning confocal microscopy as well as microscopy and rarely exceeds 4 µm (Figure S3b,c, Supporting Information).

After the flow was started, the oil drops circulated with the emulsion through the micropillar array refused to attach to the pillar walls. To test whether this was caused by electrostatic repulsion between the drops and the surface, we added different surfactants (see Sections S3.1 and S3.2, Supporting Information) to the emulsion. Indeed, after the addition of tiny amounts of the positively charged surfactant cetyltrimethylammonium bromide (CTAB, 500 µg L^−1^) drops attached to the pillars. This is in line with the observation that silicone oil‐water interfaces are negatively charged.^10^, ^11^, ^12^ The surfactant reduces the electrostatic repulsion between the oil droplets (Figures S3d,e, S4, S5, and S6, and Video S1, Supporting Information), which prevented their attachment and coalescence. Top view images demonstrate that droplets (yellowish domains) quickly begin to attach to the micropillars (encircled white dots, Figure 2b). The free emulsion droplets move too fast to be monitored. After 1.5 min almost every pillar has oil droplets attached to it (Figure 2c). As the experiment progresses, the attached drops grow in size due to coalescing with newly arriving and already attached neighboring droplets, Figure 2d–i. Eventually, the drops descend and cover the underlying solid substrate (Videos S2 and S3, Supporting Information).

3D laser scanning confocal microscopy images demonstrate that the growing drops keep their shape spherical during the whole growth process, pointing out that the Laplace pressure, Δ*P* = 2γ/*R* dominates shear‐induced deformations (**Figure** **3**a; Video S4, Supporting Information). Here, *R* is the radius of an attached oil drop and γ the interfacial tension. The green rectangles highlight that multiple drops can attach to the same pillar (Figure 3b; Figure S7, Supporting Information). Notably, micropillars can repeatedly accommodate droplets also when portions underneath the pillar are already filled (see the red and white circles in Figure 3; Figure S8, Supporting Information). This is confirmed by side view images revealing detailed information on the height and position along the pillar where droplets attach, Figure 3b,c for a sketch of the time evolution.

**Figure 3:**
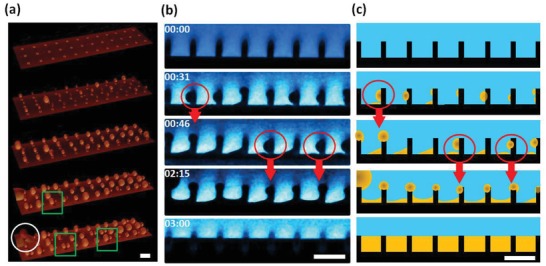
Filling of a micropillar array with lubricant. An oil‐in‐water emulsion (2 wt% of silicone oil, 500 µg L^−1^ CTAB) is circulated over the micropillar array at an average flow velocity of 77 mm s^−1^. Pillar dimensions: diameter *d* = 5 µm, center‐to‐center spacing *P* = 20 µm, and pillar height *h* = 10 µm. a) 3D laser scanning confocal images recorded in the reflection channel are visualizing the time evolution of the filling process (40×/1.11 water immersion objective lens). Multiple oil droplets can attach to the same pillar (green rectangles), and the drops can repeatedly be accommodated (white circle). For this experiment, the oil has been dyed with Coumarin 6 with subsequent image inversion. b) Laser scanning confocal side view images recorded in the fluorescence channel. Starting from an initially water‐filled micropillar array, the oil (black) gradually replaces the water (blue) in‐between micropillars (black). Red circles: Droplets just before coalescing with the oil film. Red arrows: After coalescence, newly arriving droplets attach to the same pillars. The water is dyed with 1 µg g^−1^ Atto 488 NHS‐Ester. The images give the impression that the pillars are sticking out of the lubricant phase. This false impression is caused by a combination of errors due to the mismatch of the refractive indices and a lens effect because of the top face of the pillars is not completely flat. c) Schematic illustration of the filling process. Oil is colored in yellow, water in blue, and the pillars in black. The scale bar is 20 µm.

The emulsion droplets (black in Figure 3b, yellow in Figure 3c) preferentially attach to the pillars' front side, i.e., in the flow direction (Figures S9 and S10, Supporting Information). Attached drops grow via coalescence with newly arriving droplets and neighboring drops along the pillar walls and tops. If the droplet touches the bottom surface, capillary forces pull the drop down, causing it to spread within the microstructure. The red circles highlight examples of drops just before descending. Gradually, the whole space in‐between the micropillar gets filled with oil, and a lubricant‐impregnated slippery surface is established. Note that gravity does not cause descending of drops because the silicone oil has a lower density (ρ_O_ = 0.96 g mL^−1^) than water (ρ_w_ = 1.00 g mL^−1^). After lubricating the structure by the emulsion, water drops could easily slide on the surface when the substrate was inclined by less than 10° (Video S5, Supporting Information).

Quantitative data on the attachment and filling process are obtained using xy‐image sequences acquired in the fluorescence channel. The orientation of the drops attached to the pillars concerning the flow direction of the emulsion is defined via an orientation angle (**Figure** **4**a). An orientation angle of zero means that the drops attach to the pillars precisely in the flow direction of the emulsion. The orientation angle has been measured for every drop in every frame of the image sequence. Angles vary between 0° and 180°, assuming that attachment is symmetric with respect to the flow direction. In the initial stage of the filling experiment, the first third of the experiment, over 86% of the droplets attached at angles below 45° (Figure 4b; Figures S9 and S10, Supporting Information). The inset visualizes the preferred attachment of drops in the flow direction (higher magnification shown in Figure S10, Supporting Information). As the filling progresses further, the occurrence of a preferred direction of drop attachment in flow direction is progressively lost (the successive two‐thirds of the filling experiment, Figure 4b and Figure S11, Supporting Information). Newly arriving drops preferentially attach to the pillars tops because these regions are more accessible to the flowing oil droplets. The individual drops do not change their position during their whole lifetime (Figure S12, Supporting Information). Indeed, the lateral adhesion greatly exceeds the shear‐induced depinning force (see Section S3.5, Supporting Information). For the vast majority of droplets, the projected surface area remained below 50 µm^2^ before they descend to the bottom substrate, corresponding to a drop diameter below 8 µm (Figure S13, Supporting Information).

**Figure 4:**
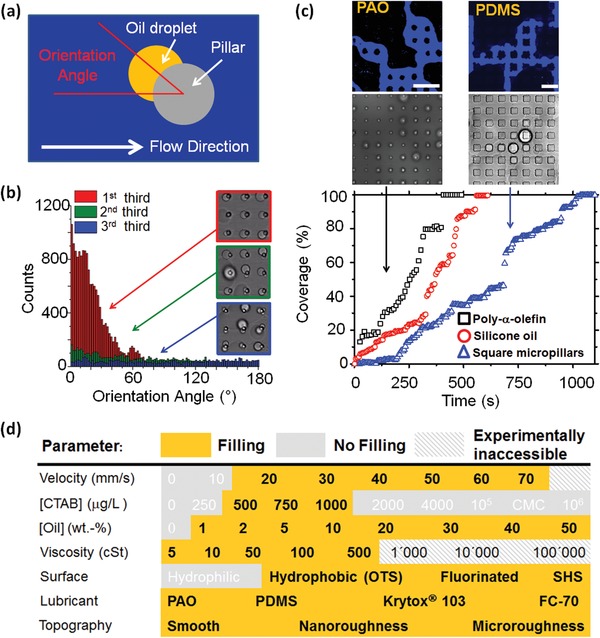
Drop orientation and filling process. a) The orientation angle is defined as the angle between the horizontal flow direction and the connecting line between the center of the projected area of the pillar and the droplet's center of mass. Drops attaching at an angle of, e.g., 10° and −10° are counted together as they can be treated to be equivalent. b) Orientation angle at the first, second, and third last of the filling process. Insets: transmission channel images of drops attached to the pillars at different phases of filling. Cylindrical pillar dimensions: diameter *d* = 5 µm, center‐to‐center spacing *P* = 20 µm, and pillar height *h* = 10 µm. c) Time evolution of the coverage for three different filling experiments using the industrially relevant poly‐α‐olefin (black squares, fluorescence channel (top) and transmission channel (bottom) snapshot of filling process on the upper left, scale bar: 50 µm) and biocompatible silicone oil with cylindrical (standard case, red circles) and quadratic (blue triangles, fluorescence channel (top) and transmission channel (bottom) snapshot of filling process on the upper right, scale bar: 50 µm) micropillars. Quadratic pillar dimensions: edge length = 20 µm, center‐to‐center spacing *P* = 40 µm, pillar height *h* = 10 µm. Apart from changing the oil and pillar, geometry and the experimental conditions were identical. d) Phase diagram showing parameter windows for successful filling experimentally verified in this work (yellow) and conditions at which successful filling is not achieved (grey). The experiments where the velocity and CTAB concentration were not varied were performed at a constant average velocity of 77 ± 2 mm s^−1^ and a CTAB concentration of 500 µg L^−1^. The different parameters can be varied independent of each other.

The temporal evolution of the filling kinetics of individual filling experiments varies as shown in Figure 4c, where the time evolutions of the oil coverage of the underlying bottom substrate are shown. Often a slowing down of the filling can be observed in the course of time (Figure S14, Supporting Information). Likely, this is caused by the accumulation of repulsive charges at the surface of oil droplets and replenished films. Coalescence requires that electrostatic repulsion between oil droplets needs to be overcome. Indeed filling works best for surfactant concentrations between 500 and 1000 µg L^−1^. For the concentration range of 500–1000 µg L^−1^ of CTAB, the surfactant molecules cover ≈0.03–0.06% of the surface area of the drops (Figure S6 and Section S3.2, Supporting Information). For increasing surfactant concentration, droplets preferentially attach at the pillars' base and the bottom substrate. For high concentrations of CTAB > 4000 µg L^−1^ (1.1% critical micelle concentration, CMC), the oil droplets in the emulsions become sufficiently positively charged that electrostatic repulsion of the charged oil‐water interface again impedes coalescence of droplets and filling of the structure (see the Supporting Information).

The filling mechanism works for a wide range of velocities, oil concentrations, and viscosities (Figure 4d). The maximum accessible velocity and viscosity were given by the setup. To investigate whether the filling works analogously for different surface structures and emulsions, we first filled arrays of quadratic pillars of larger size (edge length: 20 µm, blue triangles, Figure S15, Supporting Information). The structures can be filled, showing that the filling does not depend on details of the flow profile. The lubricant can also be replenished on fluorosilane coated micropillars and using nanostructured, porous surfaces, for example, glass coated with silicone nanofilaments^13^ (Figures S16–S18 and Video S6, Supporting Information). Also, the oil used to prepare the emulsion can be replaced. We tested the industrially widely used poly‐α‐olefin (PAO, ρ = 0.78–0.82 g mL^−1^), Figure 4c, black squares (and Figure S18a, Supporting Information), and the fluorinated oils,^2^, ^7^, ^14^, ^15^ Krytox 103 and Fluorinert FC‐70 (FC‐70), which are commonly used lubricants for slippery surfaces (Figure S19, Supporting Information). In all cases, the negative charge of the emulsion drops had to be compensated by adding 500 µg L^−1^ of CTAB to the water phase before emulsification. This also indicates that a flow‐induced separation of the less viscous component to the walls is unlikely to be the cause of the filling. Whereas PAO contains many components of greatly varying molecular weights and viscosities, silicone oil, Krytox 103, and FC‐70 that are fairly monodisperse (Figure S20, Supporting Information).

The generality of the phenomenon poses the question, whether flow‐induced but shear‐resistant lubrication is to be expected. No, because continuity of interfacial velocities *v*_i_ and shear stresses τ_xy_ across the interface take lubricant along. The shear stress induced by the flowing emulsion can be estimated from the geometry and flow rate. It is safe to assume laminar flow, since the Reynolds number (*Re* = ρ_e_ · *v* · *H*/η_e_) is 0.1 for this flow condition. Here, ρ_e_ ≈ 1 g mL^−1^ is the density of the emulsion, η_e_ = η_w_ · (1 + 5/2 · φ) is the viscosity of the emulsion, η_w_ = 1 mPa s is the viscosity of water, and ϕ is the fraction of oil present in the emulsion.^16^ As the height of the surface structures is more than an order of magnitude less than the height of the flow cell, the flow profile is parabolic through its depth, and the flow rate *Q* imposes a shear stress τ_xy_ on the top face of the micropillars:^8^, ^9^

(1) $$\tau_{\mathrm{xy}}=\;\frac{6\cdot \eta_{e}\cdot Q}{W\cdot H^{2}}$$

This yields τ_xy_ =  0.8 Pa for the model emulsion ϕ  = 0.02, η_e_ = 1.05 mPa s, and *Q* = 7 mL min^−1^. The amount of lubricant sheared off by the flow has been estimated assuming completely filled simple rectangular groove geometry. In that case, shear‐induced depletion per unit of time along single grooves can be estimated to be^9^

(2) $$q\;=\;c\frac{wh^{2}}{\eta }\tau_{\mathrm{xy}}$$

Here, the constant *c* ≈ 0.15 accounts for the hydrodynamic resistance created by the walls of the grooves and is dependent on the aspect ratio of width *w* = *P* – *d* and the height *h* of the grooves. The total shear‐induced depletion of lubricant within a flow channel made of *N* grooves is *q*_d_ =  *N* · *q*. Assuming that a row of micropillars resembles a groove the amount of sheared off lubricant can be estimated as *q*_d_ =  6.6 × 10^5^ µm^3^ s^−1^ for the model emulsion and flow channel with *w* = 15 µm, *h* = 10 µm, and *N*  =  190.

Theoretically, the hydrodynamic drag force of water (viscosity η_w_ = 1 mPa s) *F*_hyd_ should prevent coalescing of the oil drops and filling of the structure.^11^, ^17^ For spheres, $F_{\mathrm{hyd}}=\;-\frac{6\pi \eta_{w}vR^{2}}{x}$diverges if the distance *x* between the oil droplets of radius *R* and the surface approaches zero. Because drops are deformable, no analytical expression exists. Still, only at sufficiently high velocity *v* or long contact times; the water film separating the drop and the pillar is sufficiently thinned during the impact that a defect can induce rupturing of the water film.^18^ One might speculate whether diffusion of oil and nucleation of drops might give rise to faster filling. Both processes can safely be ignored because the solubility of silicone oil in water is negligible, <1 part per billion (ppb).^19^

Beside of a minimum flow rate, experimentally found to be *Q* ≈ 1 mL min^−1^ corresponding to an average flow velocity *v* ≈ 10 mm s^−1^, Figure 4d (and Figure S21, Supporting Information), good chemical compatibility of the emulsion drops and the surface is essential. Analogous to oil capture underwater,^20^ hydrophilic arrays (neat SU‐8 micropillars and plasma‐activated micropillar arrays) cannot be filled, as oils avoid hydrophilic surfaces when surrounded by water, Figure 4d (and Figure S22, Supporting Information). Different from underwater oil capture, here the oil drops are freely circulated within the surface structure of the substrate by a flow. The filling of surface structure by oil is solely based on attachment and merging of drops.

To fill the structure, the lubricant sheared off from the surface needs to be compensated by the coalescing droplets at the surface structure. This implies that the filling rate $\overset{\cdot }{V}$ has to exceed the amount of sheared off lubricant, $\overset{\cdot }{V}>q_{d}$. An upper limit of the filling rate is given by

(3) $${\overset{\cdot }{V}}_{\max}=\;Q\;\cdot \;\phi \;\cdot \;\frac{h}{H}$$

As the filling rate and the shear stress at the surface are both dependent on the flow rate *Q*, ${\overset{\cdot }{V}}_{\max}$ always exceeds the amount of depleted lubricant *q*_d_ independently on the flow velocity (Figure S23 and Sections S3.5, S3.9, and S3.10, Supporting Information).

In conclusion, lubricant‐infused surfaces can be facilitated on a broad range of solid–lubricant combinations. In contrast to all ex situ approaches, the presented in situ replenishing strategy does not rely on spraying, dipping, or painting. The dispersed phase acts as an autonomously replenishing lubricant. Also, high‐viscosity oils can be transported quickly in a diluted oil‐in‐water emulsion. The generic nature of the filling mechanism and the successful use of different lubricants demonstrate the potential for industrial applications. Basically, for every commercial product involving the flow or movement of emulsions over a porous surface, i.e., the walls of the container containing an emulsion‐based product, our approach could be applied.

## Experimental Section

*Materials*: The surfactant CTAB was purchased from Sigma‐Aldrich. trichloromethylsilane (TCMS), OTS, and fluorosilane, 1H,1H,2H,2H‐perfluorooctyl‐trichlorosilane were purchased from Sigma‐Aldrich. The SU‐8 photoresist was purchased from MicroChem Corp. The fluorescent dyes were ATTO 488 NHS‐ester (ATTO‐TEC GmbH, Germany, diluted in MilliQ water) and Coumarin 6 (Sigma‐Aldrich). As lubricants silicone oil (PDMS, Sigma‐Aldrich, viscosity: 50 cSt at 25 °C), poly‐α‐olefin (PAO, Durasyn 166, Tunap Industry, Canada), and Krytox 103 (DuPont) were used. All chemicals were used as received. The flow cells (Sticky‐Slide VI^0.4^) were ordered from ibidi GmbH. The flow cells were connected to a peristaltic pump (Reglo‐Analog MS4/8, Cole‐Parmer GmbH) via polyvinyl chloride (PVC)‐tubes (internal diameter = 1.42 mm, Novodirect GmbH Labor und Meßgeräte) and tightly sealing linkers. The fluorescent dye ATTO 488 NHS‐ester offers excellent water solubility, strong absorption, high fluorescence quantum yield, and high photostability. The fluorescence is excited most efficiently in the range of 480 and 1515 nm. The 488 nm line of the argon laser was used for excitation.^21^ To visualize replenishment of the lubricant within the nanofilament coating, Coumarin 6 with the concentration of 50 µg g^−1^ was used to label the silicone oil. The 476 nm line of the argon laser was used for excitation. The used dye concentrations did not change the interfacial tension of water. Before preparing the emulsion, the dyed oil was sonicated for 2 h and filtered through a 0.22 µm syringe filter to remove possible aggregates of the dye.

*Setup of Flow Cell Experiment*: The OTS‐coated micropillar arrays were stuck onto flow cells (Figure S1 and Table S1, Supporting Information). To ensure tight sealing a ferrule was used. The flow cell consists of 6 individual rectangular channels. The individual channels are 0.4 mm high, 17 mm long, and 3.8 mm wide, which amount to a coverable area of 64.6 mm^2^ per channel. Each channel has a volume of 30 µL. The rectangular flow cells have plastic side and top walls based on epoxy resin (Figure S1, Supporting Information). The flow cells are mounted to the sample surface by an adhesive layer, which adds 0.1 mm to the overall height of the flow channel, i.e., *H* = 500 µm. The flow cell with the affixed micropillar array was connected to PVC tubes via rectangular linkers. The tubes were fixated on a peristaltic pump and immersed into the emulsion (Figure S2, Supporting Information). By choosing the pumping speed at the peristaltic pump, the volume flow rate of emulsion flowing through the channel could be adjusted at our discretion. The volumetric flow rate established through the channel was to amount to *Q* = 7 ± 0.2 mL min^−1^, which results in an average velocity of 77 ± 2 mm s^−1^. This implies that the flow is laminar (*Re*  =  ρ_e_ · *v* · *H*/η_e_ = 0.1 < 1). Figure S2 (Supporting Information) shows a photograph of the experimental setup (top) and a schematic of the filling experiment (bottom). The flow cell setup was mounted onto the commercial laser scanning confocal microscope (Leica TCS SP8 SMD). The tubes were sufficiently long to connect to the flask containing the emulsions. Before starting the experiment with the emulsion, the channels were filled with dyed water (1 µg mL^−1^) to ensure that there are no air bubbles left and to tune the confocal image acquisition parameters. The prevenient filling of the channel with water does establish the situation of a porous structure (micropillar array) being completely devoid of lubricant, i.e., it establishes the situation of complete lubricant depletion from which we start the filling experiment. Before each experiment, the connecting tubes were rinsed multiple times with MilliQ water and dried with pressurized nitrogen. The hydrophobic PVC tubes prefer oil over water. As a consequence, the tubes can be wetted by oil and an oil drop might form. In some of the experiments, the oil droplet is taken along with the emulsion, entering the flow cell and interfering with the measurements. These data were excluded from further considerations.

*Preparation of Emulsions/Facilitating Coalescence*: A volume of 19.4 mL of MilliQ water containing a concentration of 500 µg L^−1^ of CTAB was added to 0.4 g of the silicone oil. Furthermore, 0.2 mL of MilliQ water containing the fluorescent dye at a concentration of 100 µg mL^−1^ was added so that the total concentration of the dye in the emulsion was 1 µg mL^−1^. The emulsification was done using a tip sonicator (SONIFIER W‐450D, G. Heinemann Ultraschall‐ und Labortechnik) for 2 min (70% amplitude, 20 s pulse duration, 10 s of pause between pulses). During the sonication, the emulsion was kept in an ice bath. Silicone oil droplets in an aqueous environment are negatively charged,^11^ which gives rise to a repulsive force between the drops and good stability of the emulsion. The concentration of the positively charged surfactant CTAB^10^ was chosen in such a way to allow the drops to attach and coalesce at the micropillar array still having good stability within the emulsion. The concentration of the surfactant, 1.4 × 10^−6^ m, is selected close at the point of charge reversal (pcr), orders of magnitude below the CMC of CTAB (1 × 10^−3^ m). Pendant drop and Tensiometer (Wilhelmy plate) measurements evidence that, within the experimental accuracy, the surface tension values measured at this surfactant concentration do not deviate from the value for pure water. When observed with the LSCM, it is revealed that the emulsion is very polydisperse, having oil droplet diameters up to ≈4 µm (Figure S3b,c, Supporting Information).

*Preparation of Micropillar Arrays*: The preparation of the neat/nonfunctionalized SU‐8 micropillar arrays is described elsewhere.^4^, ^22^ The regular and uniform SU‐8 photoresist micropillar arrays were fabricated on 170 µm thick coverslip glass slides (Thermo Fisher Scientific). Prior to photolithography, the glass slides were plasma‐activated (Femto, Diener electronic GmbH + Co. KG) with for 1 min (oxygen plasma, 65% of the maximum intensity of 300 W, pressure below 0.3 mbar). Subsequently, the samples were subjected to CVD at ≈200 mbar for 2 h to deposit a trichlorooctylsilane (or fluorosilane) monolayer onto the substrates (100 µL of silane together with the samples were placed in the desiccator) to render the surfaces more hydrophobic.^23^ After the CVD‐process, the samples were put into a vacuum oven (2 h, 60 °C, VTR5022, Heraeus) to get rid of residual unreacted silane and volatile contaminants.

*Preparation of Silicone Nanofilaments*: A mixture containing TCMS and *n*‐hexane (Fisher Chemical), having a water content of 85 ± 5 ppm (200 µL TCMS per 100 mL *n*‐hexane), was prepared by stirring for 60 s in a reaction container. The water content was evaluated using a Karl Fischer coulometer (Mettler Toledo C20 Compact KF coulometer). Coverslips cleaned by ultrasonication in ethanol were subsequently immersed in the solution, and the reaction container was sealed. After 2 d, the coverslips coated with silicone nanofilaments (TCMS) were rinsed with *n*‐hexane (Fisher Chemical) and dried under a nitrogen stream. More detailed information on the preparation of silicone nanofilaments can be found elsewhere.^24^

*Homogeneity of OTS‐Coating*: The homogeneity of the OTS‐coating has been confirmed by checking the static water contact angle on flat glass substrates at 24 different points for at least one sample of each batch. The static water contact angle averaged over all samples investigated amounts to 105° ± 2°. The value of the standard deviation (±2°) does not exceed the experimental accuracy of the contact angle goniometer, which proves the homogeneity of the OTS‐coating. Note that filling of the structure is not observed for the case of a hydrophilized, oxygen plasma activated, micropillar substrate. However, for the case of a fluorinated substrate, the filling mechanism works as observed for the case of OTS‐coated substrates.

*Confocal Imaging*: An inverted LSCM (Leica TCS SP8 SMD) with a 40×/1.11 water immersion objective lens (Olympus) was employed to image the oil replenishment process. The scanned areas were normally 400 × 400 µm^2^, if not otherwise mentioned. The horizontal resolution was ≈500 nm, the vertical resolution was ≈1 µm and the time span in‐between successive images was 1.29 s. The scanning frequency was 400 Hz. Images were acquired at different heights with respect to the bottom of the micropillar substrate.

*Contact Angle and Interfacial Tension Measurements*: Contact angles were measured at different solid–liquid–air/solid–liquid–liquid combinations with a standard goniometer (OCA35, DataPhysics) using the sessile drop method. Interfacial and surface tensions were measured using the contact angle goniometer (OCA35, DataPhysics) with the pendant drop method and a tensiometer (DCAT 11EC, DataPhysics) using the Wilhelmy plate method. The silicone oil–water interfacial tension γ_ow_ = 39.8 ± 0.3 mN m^−1^ does not change measurably due to the addition of CTAB at a concentration of 500 µg L^−1^ (0.15% of CMC). Silicone oil droplets on a smooth OTS‐coated surface immersed in MilliQ‐water showed a static contact angle θ = 96°, an advancing contact angle θ_a_ = 113° ± 2°, and a receding contact angle θ_r_ = 0°. The vanishing receding contact angle does indicate strong pinning of the three‐phase contact line, which means that lateral adhesion is strong and large shear forces would be required to shear off droplets from the surface. To check whether the surface is slippery, we aimed to compare the mobility of water drops before and after infiltrating the structure using the emulsion. However, it turned out to be impossible to disassemble the flow cell. Therefore, we used an OTS‐coated micropillar arrays (cylindrical pillars, diameter = 5 µm and center‐to‐center spacing = 20 µm) in open air and let an identically prepared emulsion (2 wt% of silicone oil, 500 µg L^−1^ of CTAB) flow over the micropillar array. Before the emulsion was flowing over the surface, water drops pinned to the surface (Video S5, Supporting Information). After the flow of emulsion over the structure, water drops deposited on the surface could easily slide/roll‐off the surface. 10 µL water drops started to move downward the surface as soon as the substrate was inclined by 8° ± 1° determined by the contact angle goniometer at the constant inclination rate of 1° s^−1^.

*Scanning Electron Microscopy (SEM)*: The micropillar arrays were characterized via SEM using a LEO 1530 Gemini scanning electron microscope (Zeiss, Germany). The samples were tilted to best visualize a micropillar array.

*Image Processing*: The confocal images have been evaluated and processed using the Java‐based open‐source image processing software ImageJ (Fiji) and Python scripts. The statistical evaluation and the extraction of quantitative data on the behavior of the individual droplets with the aim of further elucidating the filling mechanism in detail were the principle aims of image processing. The primary focus lied on the images and videos obtained in the fluorescence channel. Low signal‐to‐noise ratios (noise), horizontal banding in the image, and poor contrast were frequent problems which had to be dealt with in the image analysis. The poor contrast originated from the fact that, since in many of the experiments the oil has remained undyed, pillars and oil droplets both appear black in the fluorescence channel. The workflow is described shortly in the following. The image processing and evaluation started with the help of adapted built‐in Fiji plugins. At first, frames were duplicated for further evaluation. Then, the individual pillars were stacked on top of each other to simplify further processing. The areas occupied with pillars were filled with background signal. A mean filter has been applied to reduce the noisiness in the data, i.e., to smoothen the images by reducing the amount of intensity variation. Subsequently, Python scripts have been used. At first, all droplets per micropillar and frame were consolidated. Then, the centers of the pillars and the droplets have been listed. To determine the orientation of the droplets with respect to the center of the pillar toward the flow direction of the emulsion in the flow channel, the respective centers of the pillars and the droplets attached to the pillars have been listed. After correcting for the sample flow channel's rotation angle against the horizontal direction, the angles obtained between the horizontal line and the straight line connecting the centers of the pillars and the droplets, referred to as orientation angle in the following, have been recorded. The orientation angles were used to quantify the orientation of the droplets with respect to the center of the pillar toward the flow direction of the emulsion in the flow channel. These orientation angles were averaged over all frames to provide quantitative data on the average orientation of the droplets with respect to the pillars and the flow direction. Furthermore, the orientation angle immediately before to the sinking of the droplet, referred to as the last angle, has been logged. In addition to the orientation of the droplets, quantitative data on the droplet sizes (projected area) and their growth were extracted. To this end, the droplets were considered spherical and the contours of the droplets, which have been assumed to represent the equatorial circumference of the droplet considered, were recorded. The averaged distances between selected points on the contour to the center of the droplets provided the radius of the spherical droplets. The projected area of the droplet, i.e., the droplet size, was obtained with the help of elementary geometry. The droplet sizes were listed for each frame of the image sequence. The droplet size immediately before the sinking event, referred to as the last area, was of particular interest. The image processing algorithms also took into account the possibilities of droplet shrinking and detachment from pillars.

## Conflict of Interest

The authors declare no conflict of interest.

## Supporting information

Supplementary

Supplementary

Supplementary

Supplementary

Supplementary

Supplementary

Supplementary

## References

[1] A. Lafuma , D. Quéré , EPL 2011, 96, 56001.

[2] T. S. Wong , S. H. Kang , S. K. Tang , E. J. Smythe , B. D. Hatton , A. Grinthal , J. Aizenberg , Nature 2011, 477, 443. 21938066 10.1038/nature10447

[3] J. D. Smith , R. Dhiman , S. Anand , E. Reza‐Garduno , R. E. Cohen , G. H. McKinley , K. K. Varanasi , Soft Matter 2013, 9, 1772.

[4] F. Schellenberger , J. Xie , N. Encinas , A. Hardy , M. Klapper , P. Papadopoulos , H.‐J. Butt , D. Vollmer , Soft Matter 2015, 11, 7617. 26291621 10.1039/c5sm01809a

[5] a) B. R. Solomon , K. S. Khalil , K. K. Varanasi , Langmuir 2014, 30, 10970; 25144426 10.1021/la5021143

[6] a) J.‐H. Kim , J. P. Rothstein , Exp. Fluids 2016, 57, 81;

[7] C. Howell , T. L. Vu , C. P. Johnson , X. Hou , O. Ahanotu , J. Alvarenga , D. C. Leslie , O. Uzun , A. Waterhouse , P. Kim , M. Super , M. Aizenberg , D. E. Ingber , J. Aizenberg , Chem. Mater. 2015, 27, 1792.

[8] J. S. Wexler , I. Jacobi , H. A. Stone , Phys. Rev. Lett. 2015, 114, 168301. 25955076 10.1103/PhysRevLett.114.168301

[9] I. Jacobi , J. S. Wexler , H. A. Stone , Phys. Fluids 2015, 27, 082101.

[10] a) Y. Gu , D. Li , Colloids Surf., A 1998, 139, 213;

[11] H.‐J. Butt , M. Kappl , Surface and Interfacial Forces, WILEY‐VCH Verlag GmbH & Co, Weinheim, Germany 2010.

[12] J. K. Beattie , A. M. Djerdjev , G. G. Warr , Faraday Discuss. 2009, 141, 31. 19227349 10.1039/b805266b

[13] F. Geyer , C. Schönecker , H.‐J. Butt , D. Vollmer , Adv. Mater. 2017, 29, 1603524. 10.1002/adma.201603524 27896855

[14] P. S. Brown , B. Bhushan , J. Colloid Interface Sci. 2017, 487, 437. 27814555 10.1016/j.jcis.2016.10.079

[15] a) N. Vogel , R. A. Belisle , B. Hatton , T. S. Wong , J. Aizenberg , Nat. Commun. 2013, 4, 2167; 10.1038/ncomms3176 23900310

[16] a) G. I. Taylor , Proc. R. Soc. A 1932, 138, 41;

[17] a) W. Hardy , I. Bircumshaw , Proc. R. Soc. A 1925, 108, 1;

[18] a) F. Mugele , B. Bera , A. Cavalli , I. Siretanu , A. Maestro , M. Duits , M. Cohen‐Stuart , D. van den Ende , I. Stocker , I. Collins , Sci. Rep. 2015, 5, 10519; 26013156 10.1038/srep10519 PMC4444960

[19] D. Graiver , K. W. Farminer , R. Narayan , J. Polym. Environ. 2003, 11, 129.

[20] M. Jin , J. Wang , X. Yao , M. Liao , Y. Zhao , L. Jiang , Adv. Mater. 2011, 23, 2861. 21538597 10.1002/adma.201101048

[21] Atto 488 NHS‐ester data sheet, Recommended Procedures for Labeling, 2017.

[22] P. Papadopoulos , L. Mammen , X. Deng , D. Vollmer , H.‐J. Butt , Proc. Natl. Acad. Sci. USA 2013, 110, 3254. 23382197 10.1073/pnas.1218673110 PMC3587223

[23] a) J. Sagiv , J. Am. Chem. Soc. 1980, 102, 92;

[24] a) G. R. J. Artus , S. Jung , J. Zimmermann , H. P. Gautschi , K. Marquardt , S. Seeger , Adv. Mater. 2006, 18, 2758;

