# Supplementary material for: Flow‐Induced Long‐Term Stable Slippery Surfaces
Source: Adv Sci (Weinh). 2019 Apr 7;6(11):1900019. doi: 10.1002/advs.201900019 (PMC6548950; doi:10.1002/advs.201900019)
Supplement: Supplementary file 1 — Supplementary [file ADVS-6-1900019-s002.pdf]

Copyright WILEY-VCH Verlag GmbH & Co. KGaA, 69469 Weinheim, Germany, 2019.

ADVANCED SCIENCE Open Access

Supporting Information

for *Adv. Sci.,* DOI: 10.1002/advs.201900019

Flow-Induced Long-Term Stable Slippery Surfaces

*Philipp Baumli, Hannu Teisala, Hoimar Bauer, Diana Garcia-Gonzalez, Viraj Damle, Florian Geyer, Maria D'Acunzi, Anke Kaltbeitzel, Hans-Jürgen Butt, and Doris Vollmer**

Supporting Information

Flow-Induced Long-Term Stable Slippery Surfaces

Philipp Baumli[†]*, Hannu Teisala*[†]*, Hoimar Bauer, Diana Garcia-Gonzalez, Viraj Damle, Florian Geyer, Maria D´Acunzi, Anke Kaltbeitzel, Hans-Jürgen Butt, and Doris Vollmer**

Contents

S1. **Flow Cell Specifications and Main Experimental Setup** (Figure S1 and S2, Table S1)

S2. **Supplementary Videos** (Videos S1-S5)

S3. **Additional Measurements/Supplementary Experiments**

S3.1 **Charging of Oil Droplets** (Figure S3, S4, and S5)

S3.2 **Influence of Surfactant Concentration** (Figure S6)

S3.3 **Attachment of Oil Droplets** (Figure S7 and S8)

S3.4 **Orientation of Droplets with Respect to the Micropillar and Size of Descending Droplets** (Figure S9, S10, S11, S12 and S13)

S3.5 **Lateral Adhesion versus Shear-Induced Depinning**

S3.6 **Slowing Down of Filling** (Figure S14)

S3.7 **Quadratic Pillars** (Figure S15)

S3.8 **Filling of Porous Substrates of Varying Geometry and with Different Oils** (Figure S16, S17, S18, S19 and S20)

S3.9 **Generic Nature of Flow-Induced Lubricant-Replenishment from Emulsions** (Figure S21, S21 and S23)

S3.10 **Hydrodynamic Drag Force**

S1. Flow Cell Specifications and Main Experimental Setup

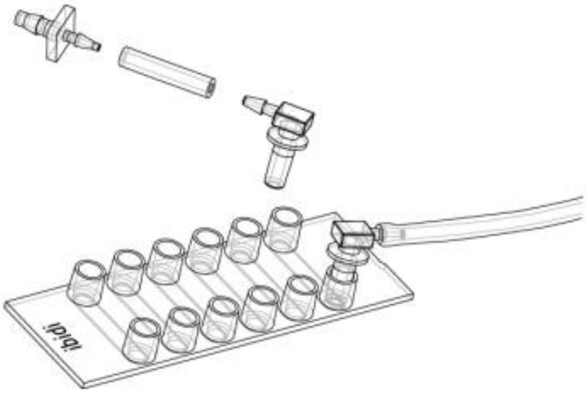

Figure S1. The flow cells used throughout this work. The technical specifications of the flow cells are presented in Table S1. (© ibidi GmbH)

Table S1. Technical specifications of the flow cells**.**

Volume per reservoir	60 μL
Number of channels	6
Volume of each channel	30 μL
Height of channels	0.4 mm
Length of channels	17 mm
Width of channels	3.8 mm

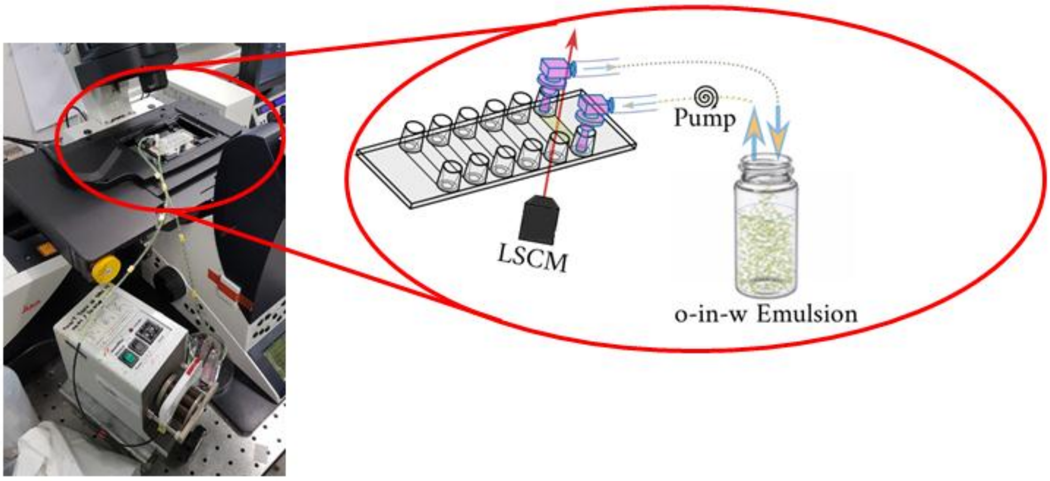

Figure S2. A photograph of the experimental setup (top) and a schematic of the filling experiment (bottom).

S2. Supplementary Videos

This section contains a list of videos (.avi).

Video S1: Silicone oil droplets in aqueous environment refusing to coalesce even if the contact line gets deformed considerably.

Video S2: Top view of filling of micropillar structure.

Video S3: Side view of filling of micropillar structure.

Video S4: Early stages of filling experiment in 3D.

Video S5: Lubricating micropillar structure by flow of emulsion in open air. The sample was inclined by 15°.

Video S6: Lubricant-replenishment on a nanofilament coating depleted with lubricant.

S3. Additional Measurements/Supplementary Experiments

S3.1 Charging of Oil Droplets

The conceptual idea behind the addition of a positively charged surfactant is illustrated in Figure S3. The emulsions remain sufficiently stable over the time scales of hours to days, also upon the addition of positively charged surfactant. In Figure S3a we present photographs of the emulsions taken immediately after preparation, 1h after preparation, 5h after preparation, and 24 after preparation. In both cases (without CTAB and with CTAB), the emulsions remain turbid. Visual inspection hardly reveals any differences. In Figure 3b and 3c, it is shown that the polydisperse character of the emulsion not containing CTAB (Figure S3b) is preserved upon the addition of CTAB (Figure S3c). Both, negative as well as positive charges on the oil droplets do stabilize an emulsion (Figure S4)[11, 25]. Addition of the cationic surfactant reduces the effective charge of the droplet, resulting in a decrease of electrostatic repulsion. As long as the charge repulsion between the charges on the oil droplet does not become too pronounced, the filling of the structure can progress. If no surfactant is added, the emulsions are very stable, but no attachment and coalescence on the solid is observed (Figure S5).

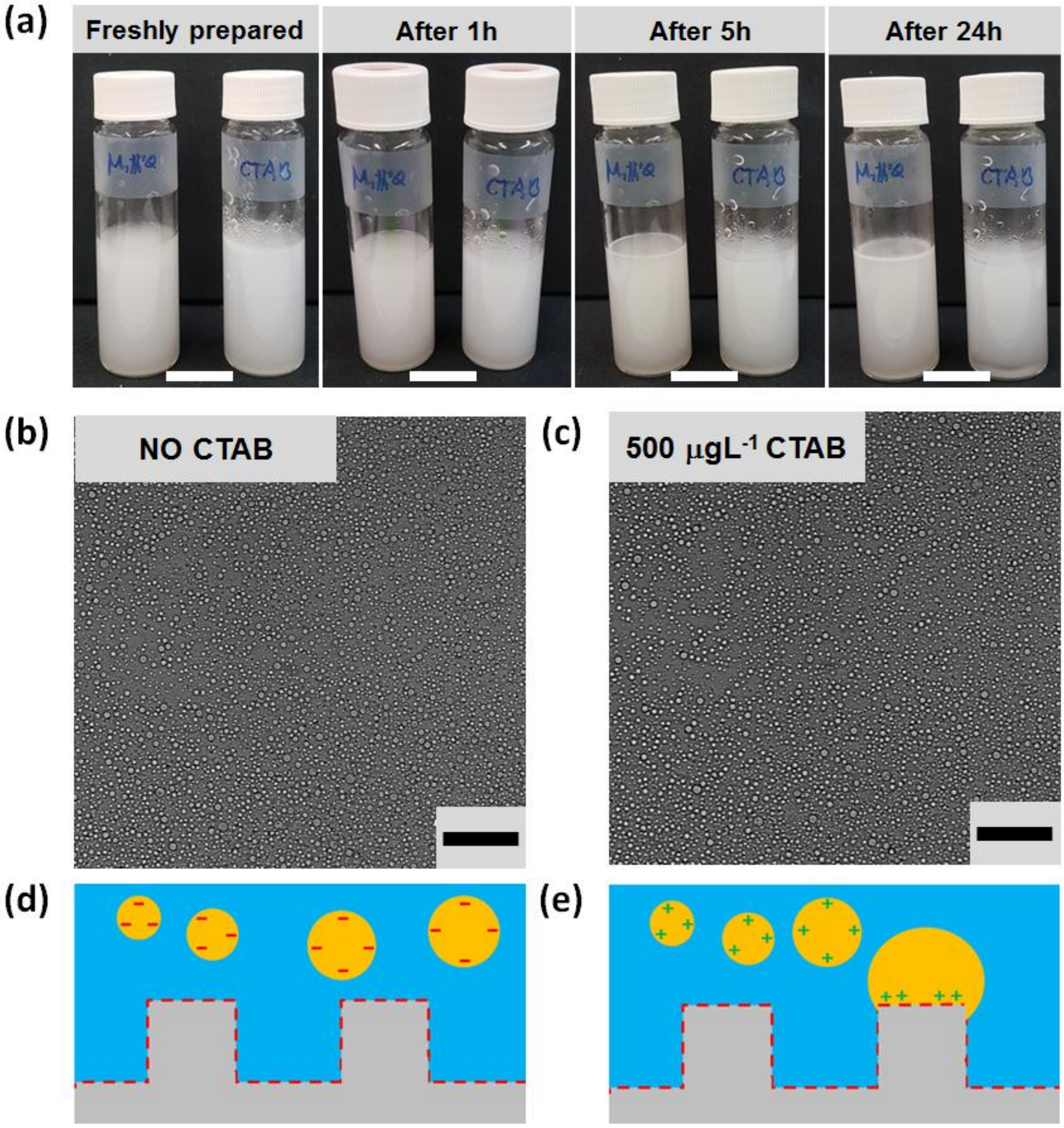

Figure S3. Negatively charged oil droplets. a) Visual inspection of emulsion stability. The emulsions remain stable long beyond the time scale of the filling experiments conducted in this work since turbidity is maintained. The scale bar is 2 cm. b) and c) Representative micrographs recorded in the transmission channel (40x/1.11 water immersion objective) showing the polydispersity of the oil-in-water emulsion c) not containing CTAB. and c) containing 500 μgL^{-1} of CTAB. Scale bar: 50 µm. d) Negative charges on the oil droplets lead to charge repulsion and no oil film formation, and filling takes place. e) Weakly charged droplets lead to attachment of oil droplets on the pillars and the bottom substrate and their growth via coalescence with newly arriving droplets, eventually leading to the filling of the structure with oil.

Figure S4 and Video S1 show that without the addition of positively charged surfactant (CTAB) the negative charges on the oil droplets are sufficiently strong to prevent coalescence in an aqueous environment even when the droplets are pushed into one another.

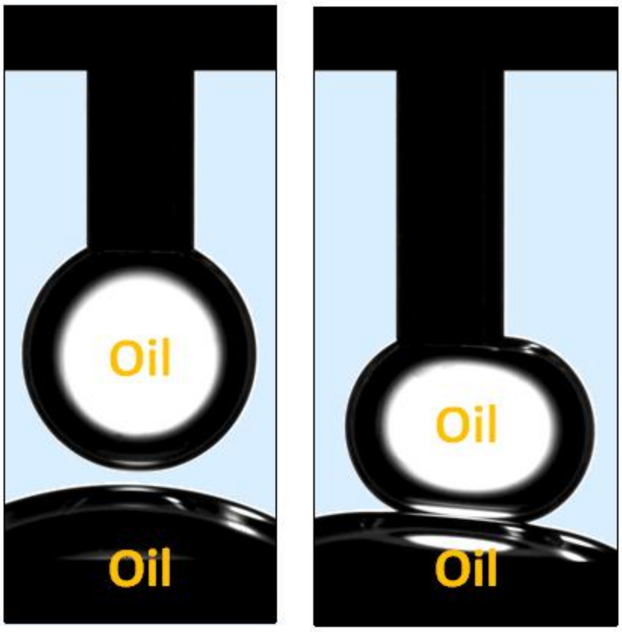

Figure S4. Negatively charged oil droplets in aqueous environment preferring deformation over coalescence.

At the later stages of the filling experiment; the filling process does slow down (Figure S14). We expect that this is due to progressive accumulation of charges at the surface of the deposited oil film, which eventually leads to charge repulsion ending the filling process. The addition of a negatively charged surfactant (SDS, sodium dodecyl sulfate) and neutral surfactant Pluronic® F-127 is not effective since it does not decrease the electrostatic repulsion between the emulsion drops.

Since a suitable content of a cationic surfactant has been added to reduce the electrostatic repulsion between the drops, the coalescence of droplets at the micropillar surface and thus formation of droplet bridges from pillar tops over the pillar walls to the bottom of the substrate depends on the flux of newly arriving drops. The flow of the emulsion facilitates the transport of the oil droplets.

The primary size or the size distribution of the arriving droplets is not relevant since the success of the filling process does not depend on either of them. The key element to the successful lubricant replenishment is the amount of cationic surfactant that is used. Also, surfaces in aqueous environments are typically negatively charged[11]. The interaction between the oil drops and the micropillar is the first event in any filling experiment. However, negatively charged oil droplets and a negatively charged surface lead to charge repulsion and droplet attachment to the micropillar array is not observed. After addition of 0.14% CMC of CATB, the emulsions are still stable for hours, *i.e.* enough for the timespan of the filling experiments which is typically ~1 h. If the concentration of CTAB is too high (>1000 μg L^{-1}), there is only a thin oil film formed on the surface with very little subsequent coalescence of oil droplets since the charge density of positive charges becomes too high. In this case, the charge repulsion between the positive charges in the oil droplets dominates and hence prevents the filling.

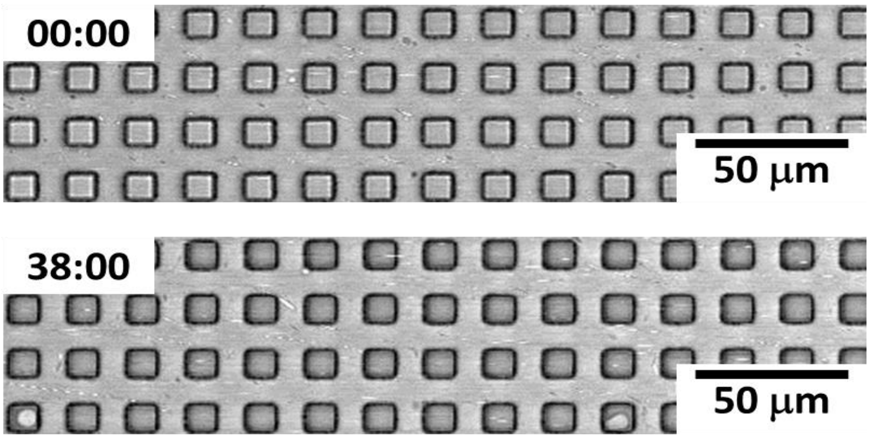

Figure S5. Without the addition of a surfactant, electrostatic repulsion between oil droplets prevents filling of the micropillar array. Even after 38 minutes (bottom) the spaces between the pillars remained water filled with no coalescence of oil droplets and filling taking place.

An upper limit of the number of CTAB molecules per drop can be estimated as follow: The molecular weight of CTAB is $M_w = 364.45$ g mol^{-1}. For a CTAB concentration = 500 µg L^{-1} (1.4 µM) this corresponds to $x = 8.4 \cdot 10^{17}$ molecules per liter. Assuming an average radius of R = 2 µm for the oil drops, the number of drops per liter of emulsion $N_d = \frac{0.02}{V_d} = 6.6 \cdot 10^{13}$, where 0.02 is the volume fraction of oil in the emulsion and $V_d = \frac{4\pi R^3}{3}$ is the volume of one drop. Thus, each drop is covered by $\frac{x}{N_d}$ = $12.7 \cdot 10^3$ surfactant molecules. Full coverage of the surface of oil drops by CTAB $\frac{A_{drop}}{A_{CTAB}} = \frac{4 \cdot \pi \cdot R^2}{A_{CTAB}}$ would imply $5 \cdot 10^7$ molecules per drop assuming area per single CTAB molecule[26] $A_{CTAB} \approx 1\ nm^2$. In reality, the number of CTAB molecules per drop will be less because not all the surfactant molecules go to the drop surface but also to the water/air interface and walls of the vessel during the preparation of the emulsions.

S3.2 Influence of Surfactant Concentration

The standard CTAB concentration used in this work (500 μg L^{-1}), an amount of 2 wt.-% oil and assuming an average radius of R = 2 μm for the oil drops, each drop is covered by less than $13{\cdot}10^3$ surfactant molecules. Full coverage of the drop surface by CTAB would imply $5{\cdot}10^7$ molecules per drop assuming each CTAB molecule covers[26] ≈ 1 nm^2. Hence, the surfactant molecules cover less than 0.03% of the surface area of the drops.

Among the surfactant (CTAB) concentrations used in this work, the concentration of 500 μg L^{-1} (≈ 1.4 μM, 0.14% of critical micelle concentration CMC = 1 mM ≈ $3.35{\cdot}10^5$ μg L^{-1}) led to successful filling of the structure. In this work, we investigated several CTAB-concentrations. In addition to the selected CTAB-concentration of 500 μg L^{-1} we worked with CTAB-concentrations of 250 μg L^{-1} (≈ 0.7 μM, 0.07% CMC), 1000 μg L^{-1} (≈ 2.7 μM, 0.3% CMC), 4000 μg L^{-1} (≈ 11 μM, 1.1% CMC), 10^5 μg L^{-1} (≈ 274 μM, 27.4% CMC), and 10^6 μg L^{-1} (≈ 2.74 mM, 274% CMC). As the surfactant concentration exceeds the concentration of 500 μg L^{-1}, the stability of the emulsions increases (less coalescence). As the surfactant concentration increases above 500 μg L^{-1}, droplet attachment to pillars is less prominent, but droplet attachment to the bottom substrate dominates. Droplet coalescence and partial filling of the structure does occur from the bottom substrate. In contrast to the case of a surfactant concentration of 500 μg L^{-1}, droplet attachment to pillars, droplet growth on pillars and descent of droplets to the bottom substrate is no longer observed. Complete filling of the structure is no longer observed. These results indicate good spreading of the oil on the substrate with increased CTAB concentrations, however, positive charge accumulation in the oil film ends the filling process. For CTAB-concentrations above 4000 μg L^{-1} droplet attachment to the bottom substrate gradually reduces. Above a concentration of 10^5 μg L^{-1} hardly any droplets still attach to the bottom substrate. No droplet attachment at all is observed above the CMC. This is expected to happen due to the surfactant accumulation at

the solid substrate, thus preventing attachement of any surfactant covered oil drops. Figure S6 summarizes our observations.

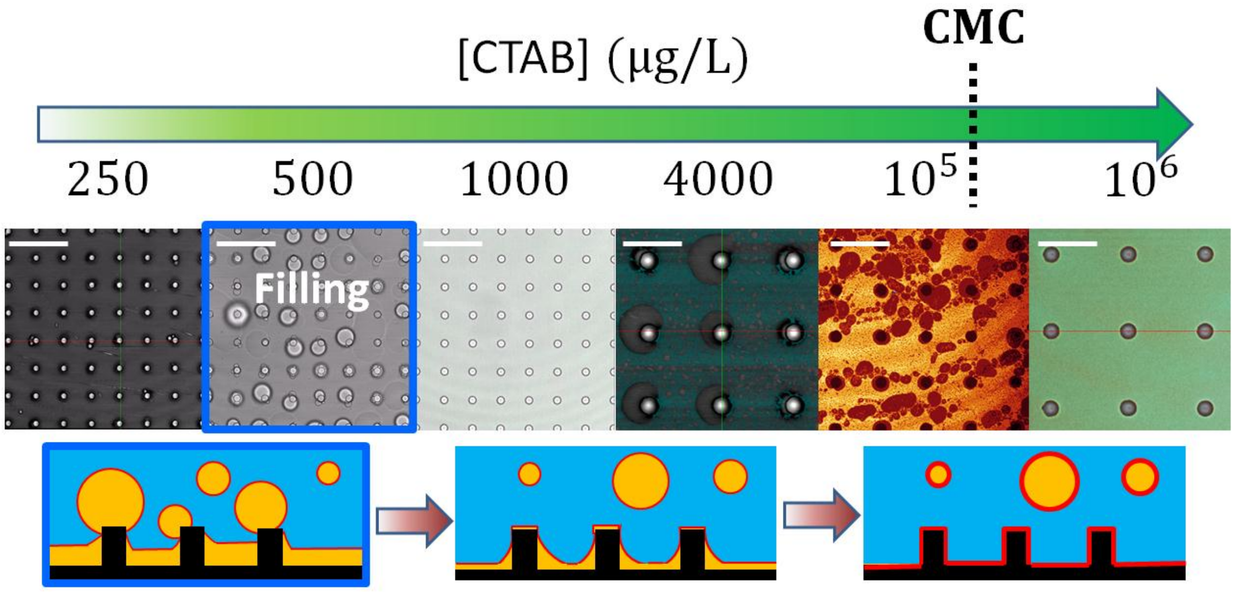

Figure S6. Filling mechanisms in dependence on the concentration of the positively charged surfactant CTAB. The concentration of 500 μg L^{-1} enables successful filling of the structure. A concentration below 500 μg L^{-1} does not overcome the charge repulsion between the negatively charged oil droplets. Hence, no coalescence and consequently no droplet attachment and droplet growth is possible. Above concentrations of 500 μg L^{-1} which remain well below the CMC of CTAB (1 mM) droplet attachment predominantly takes place on the bottom substrate, and the structure gets partially filled starting from the bottom substrate, but droplet attachment at pillars and droplet growth on pillars does no longer take place. As soon as an oil layer is formed on the bottom substrate, which may also embrace the pillars, the filling stops due to charge repulsion and increased emulsion stability. The addition of more CTAB above a concentration of 500 μg L^{-1} has led to the increased repulsion of positive charges, which dominates upon further increasing the CTAB-concentration. For CTAB concentrations approaching and exceeding the CMC, no droplet attachment is observed anymore since in these cases the strong presence of CTAB leads to strong charge repulsion

between the individual oil droplets as well as between the oil droplets and the bottom substrate. Since surfactant is always present on the bottom substrate, the strong presence of CTAB leads to a positively charged surface which repels the now positively charged oil droplets.

S3.3 Attachment of Oil Droplets

As can be seen in Figure 3a multiple oil droplets can attach to the same pillar and repeatedly accommodate droplets even after some portions of the underlying bottom substrate around a pillar have already been filled with oil. Figure S7 and S8 show magnified versions of the area marked by green squares and a white circle in Figure 3a.

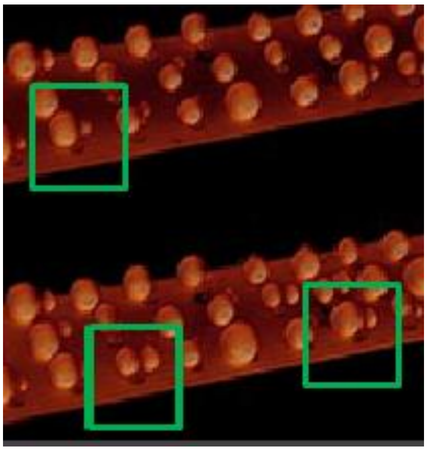

Figure S7. Magnification of the area encircled by a square in Figure 3a. Two droplets can attach to the same pillar.

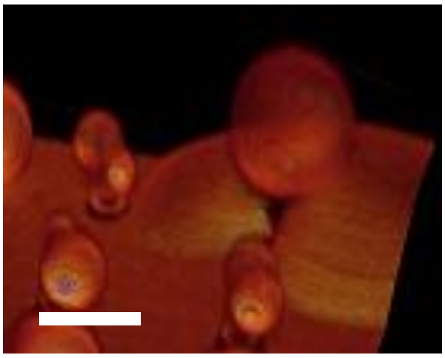

Figure S8. Magnification of the area encircled by a white circle in Figure 3a. A micropillar repeatedly accommodates a growing droplet. Portions around the micropillars are already surrounded by oil. The scale bar is 10 μm.

S3.4 Orientation of Droplets with Respect to the Micropillar and Size of Descending Droplets

At the early stages of the filling procedure, droplets are oriented towards the flow direction, as shown in Figure S9, S10, and S11. Lifetime of drops and their growth is presented in Figure S12 and S13.

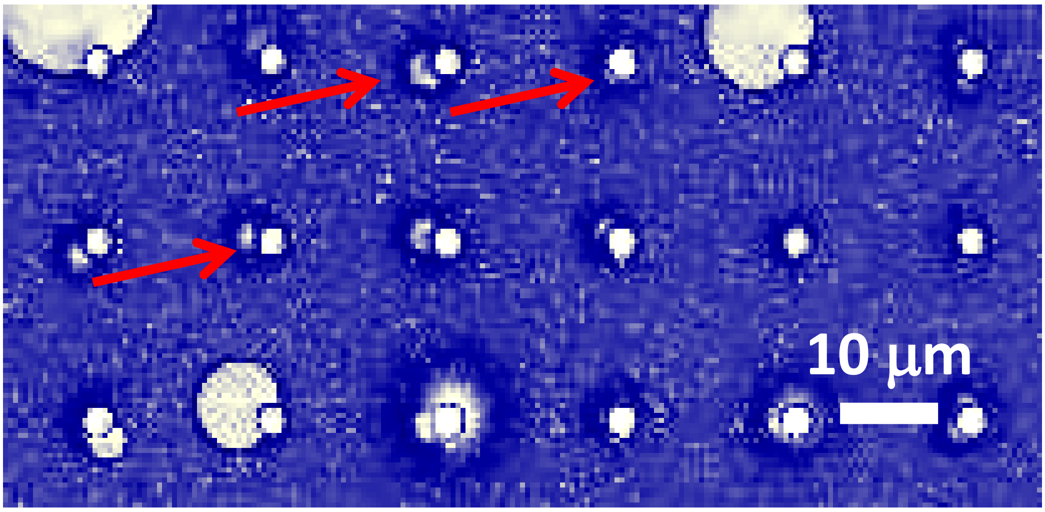

Figure S9. Droplets preferentially attach at the front side of the micropillars. Blue: water; light yellow: oil. The cylindrical micropillars are colored in white. They can be discriminated from the oil by the regular arrangement.

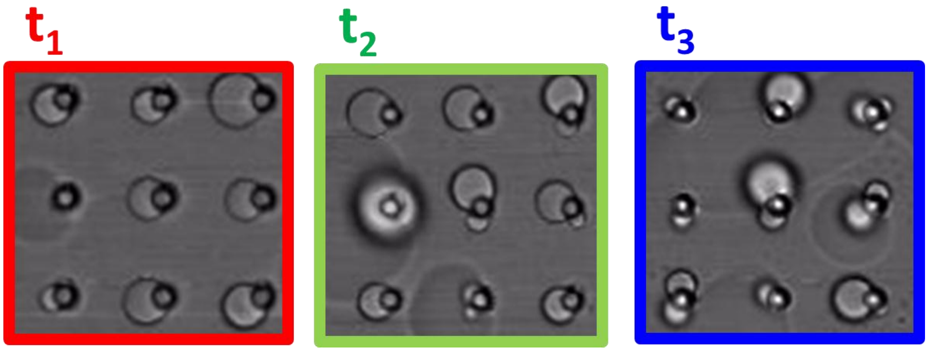

Figure S10. Magnified view of the orientation of droplets with respect to the pillars. t_1: First third of the filling (after 5.3 min.), t_2: second third of the filling (10.6 min.), t_3: last third of the filling procedure (15.9 min.). Pillar diameter = 5 µm and center-to-center spacing = 20 µm.

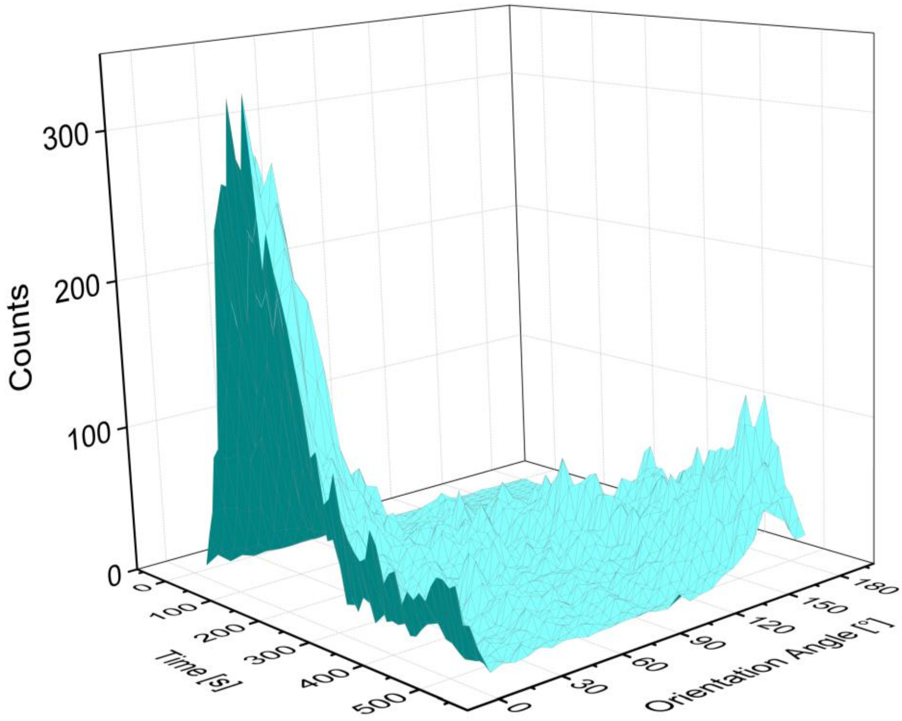

Figure S11. Temporal development of the orientation of attached droplets with respect to the center of the pillar. The majority of droplets attached to pillars are found facing the flow direction of the emulsion.

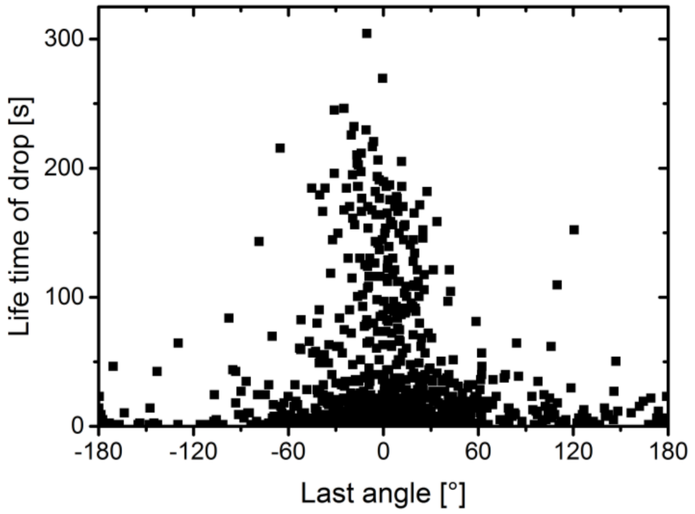

Figure S12. Orientation of the droplet just before it sank down with respect to the center of the pillar. Droplets oriented in flow direction stay on the pillars the longest before they sink down.

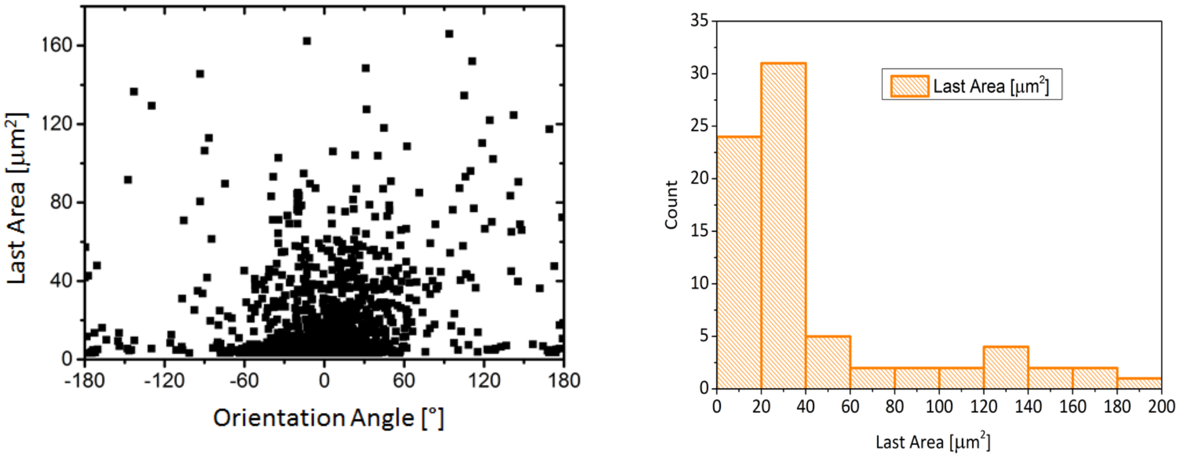

Figure S13. Left: The projected area of the droplets before they sink to the pillar substrate and their corresponding final orientation (last angle). Droplet attachment on the portions of the pillars turned away from the flow of emulsion is less frequent than droplet attachment in the flow direction. Right: The projected area to which each of the droplets had grown before it sank to the bottom substrate, the so-called *last area* is given. The data recorded in the fluorescence and transmission channel corresponding to the projected area 9 µm above the bottom substrate. The majority of droplets grew to sizes not exceeding 50 µm^2. There is no

preferred droplet size to which the droplets grow before descending to the bottom substrate. Towards the end of the filling procedure droplets primarily attach at the pillars' top faces. Now, the center of the droplets can be to the left or right of the center of the pillars, reflected in an orientation angle larger than 90°.

S3.5 Lateral Adhesion versus Shear-Induced Depinning

After attachment of a single oil drop to a pillar wall, continuity of shear stresses across the interface might remove the droplet from the wall. However, after attachment, the droplets do not leave the pillar nor do not change their position. This implies that the lateral adhesion force needs to overcome the depinning force and the shear force.

The lateral adhesion force of attached oil drops on pillar walls[27] can be estimated as $F_{adh} = k \cdot L \cdot \gamma_{ow} \cdot (\cos\theta_r - \cos\theta_a)$, where $k \approx 1$ is a dimensionless factor accounting for the precise shape of the solid-liquid-oil three-phase contact line of the drop. The drop contact width is designated by L, $\theta_a = 113 \pm 2°$ is the advancing contact angle and $\theta_r = 0°$ is the receding contact angle of silicone oil in water on smooth OTS coated SU-8 surface. Assuming a drop contact width of 5 μm (representing the width of a pillar) and the value of unity for the dimensionless factor k, the lateral adhesion force can be estimated to be $F_{adh} = 1 \cdot 5 \cdot 10^{-6}\, m \cdot 38.9 \cdot 10^{-3} \frac{N}{m} \cdot (1 - \cos(113°)) \approx 2.7 \cdot 10^{-7}$ N. The adhesion force per area is then: $F_{adh}/\pi R^2 \approx 1.4 \cdot 10^4$ Pa. This value needs to be compared to the shear force $\tau_{xy} \approx 0.8$ Pa acting at the surface (Equation (1) in main text), which is four orders of magnitude lower.

The shear stress required to depin a sessile droplet from a flat surface surrounded by an immiscible fluid is estimated by[9, 28]:

$$\tau_{depin} = \frac{\gamma_{ow}}{R}(0.28)\theta_a^{4/3}(\theta_a - \theta_r). \quad \text{(S1)}$$

With R = 2.5 µm for the small and R = 50 µm for the large droplets, the required yield stress would amount to $7.6 \cdot 10^4$ Pa and $0.4 \cdot 10^4$ Pa, respectively, more than three orders of magnitude larger than the flow induced shear stress τ_{xy} at the surface. Thus, consistently with the experiments, the calculations suggest that the droplets remain in place after attaching the pillar walls.

S3.6 Slowing Down of Filling

Without the addition of a surfactant, the underlying structure will not be filled with oil. Likely, accumulation of the surfactant molecules in the oil film causes the slowing down of the filling process with time (Figure S14).

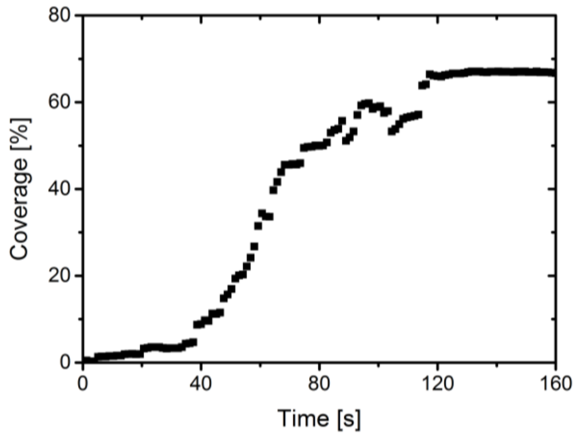

Figure S14. Temporal development of the filling of a micropillar array: the coverage saturates after 2 minutes.

S3.7 Quadratic Pillars

As a next step, the droplet distribution (number of droplets) around big rectangular pillars (40 μm edge length) has been elucidated to compare it to the droplet orientation found for the situation of cylindrical pillars (see main text, Figure 4a, b). The definition of an *orientation angle* is not appropriate for the case of rectangular pillars owed to the presence of sharp edges. In Figure S15 the time evolution of the droplet distribution is presented for a filling experiment conducted with a micropillar array consisting of large rectangular pillars. The standard experimental conditions were established (see main text). Owed to geometry three cases need to be distinguished. There is no radial symmetry. The side of the rectangle facing the flow direction (black curve, in Figure S15), the side turned away from the flow direction (blue curve, in Figure S15) and the two equivalent sides parallel to the flow direction (red curve, in Figure S15). Analogous to the case of the cylindrical micropillar arrays, the number of attached droplets is larger in the early stage of the filling experiment. As more and more portions of the bottom substrate get filled, the number of recorded attached droplets decreases. The three curves show very similar disposal. The disposal of the curves is connected to the filling kinetics of the individual experiment, which may vary significantly for individual filling experiments performed under identical conditions. There is no preferred orientation for droplet deposition.

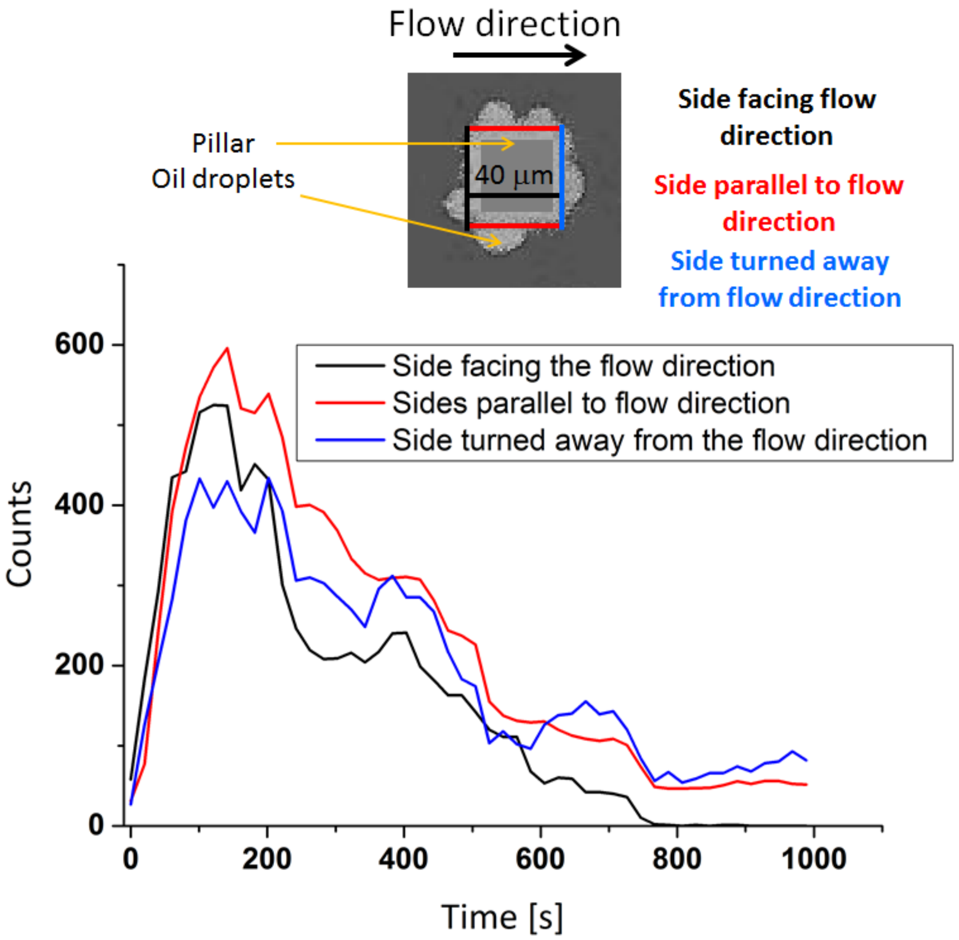

Figure S15. Time evolution of droplet distribution around large rectangular micropillars (40 µm side length) during a filling experiment (flow direction from left to right). Three cases need to be distinguished (see sketch on top): the side facing the flow direction (black), the side turned away from the flow direction (blue) and the two sides parallel to the flow direction being equivalent (red). The three curves show very similar disposals. The side facing the flow direction is not preferred over the side turned away from the flow direction regarding more frequent drop attachment.

S3.8 Filling of Porous Substrates of Varying Geometry and with Different Oils

The approach used in this work can potentially be extended to various other porous substrates different from micropillar substrates. To find out whether the mechanism also applies to nanoscopic, porous structures and whether lubricant can be replenished, we investigated glass coated with silicone nanofilaments[13], Figure S16. Silicone nanofilaments consist of a fibrous structure of filaments having a diameter of approximately 70 nm.

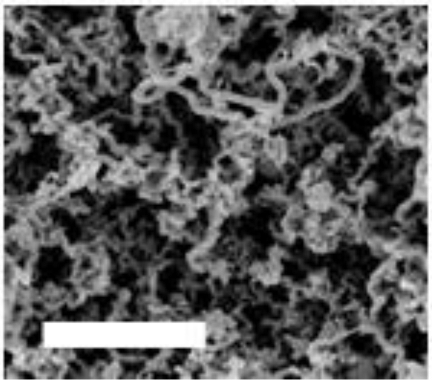

Figure S16. SEM image of a glass substrate coated with silicone nanofilaments (Scale bar = 3 μm).

In Figure S17, top row, in the fluorescence channel images the level from which the fluorescence signal from the oil-containing dyed water (blue) can be detected changes within the course of the experiment (left: beginning of the filling experiment, right: end of filling experiment), which indicates that the porous substrate gets infiltrated with the emulsion. In Figure S17, on the middle row, in the reflection channel images, the large refractive index mismatch leads to pronounced reflection signal at the beginning of the filling experiment (left) since pores in-between nanofilaments are void of oil or water and only contain air. At the end of the filling experiment (right), the pores are filled with the emulsion. Hence, the reflection signal decreases due to the less pronounced refractive index mismatch, which demonstrates the generality of the filling mechanism.

Figure S17, bottom row, reveals lubricant replenishment on partially oil-depleted nanofilaments coating (SEM image on the left). On the middle parts overlays of laser scanning confocal images consisting of images recorded in the reflection channel and the fluorescence channel are presented. The nanofilament-coated glass slide stuck onto the flow cell and pre-filled with undyed silicone oil. The amount of oil was then reduced by blowing lubricant away using a flow of nitrogen (middle left). The filling experiments were conducted with an emulsion containing silicone oil dyed with a hydrophobic fluorescent dye Coumarin 6 and the surfactant. As the filling experiment progresses, more and more dyed oil coming from the flow of the emulsion replenishes the depleted lubricant leading to a bright yellow signal corresponding to the replenished oil in the fluorescence channel (middle right, sketch on the right, Supplementary Video S5).

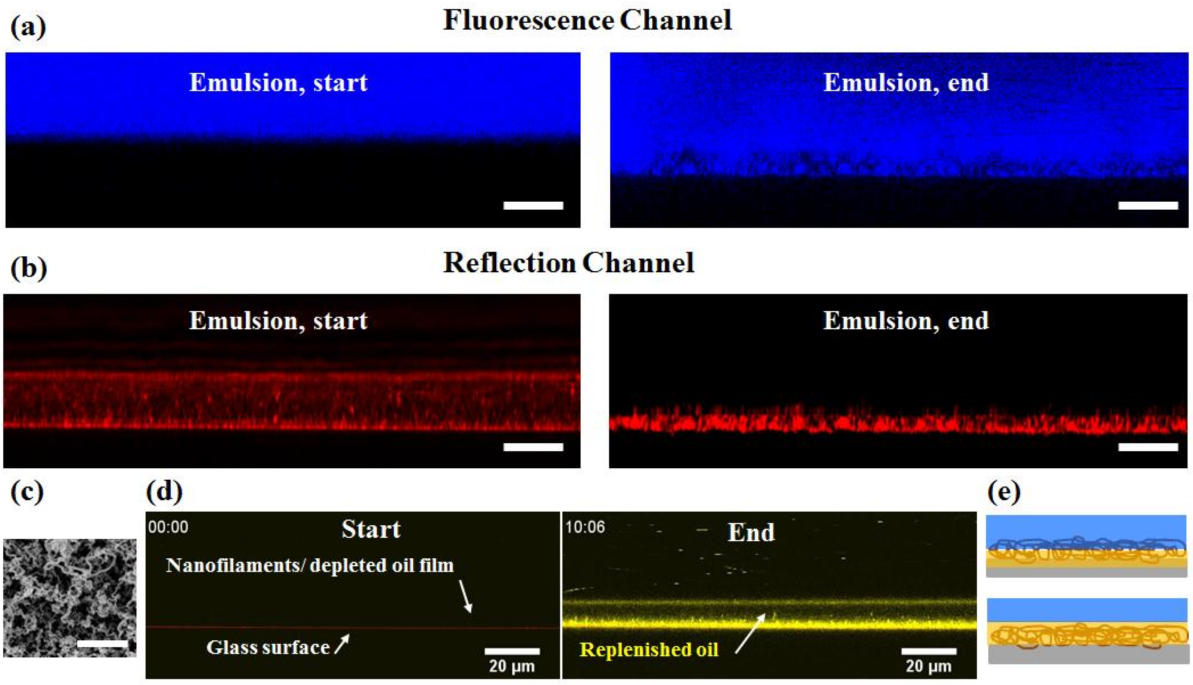

Figure S17. Filling of a porous and unordered nanofilament coating with emulsion. a): fluorescence channel (water in blue, oil undyed, scale bar is 20 µm), b): reflection channel

(scale bar is 20 µm). c) SEM image of the nanofilament coating, scale bar is 3 µm. d) depleted oil film in nanofilament coating on a glass substrate (left) can be replenished with silicone oil (dyed yellow, right). The laser scanning confocal microscopy side view images consist of an overlay of the reflection and fluorescence channel. At the beginning of the experiment, only the reflection signal owed to the mismatch in the refractive indices of emulsion/filament/oil/glass substrate interfaces are shown. Within the course of the experiment, the depleted oil layer (not dyed in the left part of b)) within the nanofilament coating gets replenished with fresh oil (dyed yellow in the right part of part b)) from the emulsion. The bright fluorescence signal at the top of the oil film and at the glass substrate is due to a tendency of the dye to accumulate at the interfaces. e) Sketch of the start and end of the experiment.

So far only silicone oil was used. This poses the question whether the filling mechanism works analogously for different lubricating oils. Figure S18a shows the filling of a micropillar structure with the lubricant poly-α-olefin (PAO, ρ = 0.78-0.82 g mL^{-1}). The key strength of PAO lies in its compatibility with many essential lubricant additives, such as antioxidants, anti-wear additives, anti-corrosion agents, friction modifiers or viscosity modifiers[29]. Again, 500 µg L^{-1} CTAB was added to the water phase before emulsification (2 wt.-% of PAO). The top row shows the fluorescence channel (water in blue, oil and pillars in black) and the bottom row the corresponding image in the transmission channel. The filling kinetics resembles those observed for silicone oil. Also, with a fluorinated lubricant Krytox® 103[7, 15, 30], which is a commonly used lubricant for slippery surfaces, the same mechanism of droplet attachment to the pillars walls, growth, coalescence and descending by droplet-bridging was observed (Figure S19). Analogously to the case of using silicone oil as lubricating oil, filling of the

structure with PAO and Krytox® 103 did not occur without added surfactant, hinting that PAO and Krytox emulsions are negatively charged.

To test the generic nature of flow-induced filling, we investigated the influence of the surface structure. Figure S18b shows the top view of the time evolution of a pillar array consisting of square pillars (edge length: 20 μm, center-to-center spacing: 40 μm, pillar height: 10 μm). Again, the top row depicts the fluorescence channel, and the bottom row the transmission channel. The transmission channel image sequence demonstrates that the droplet growth and coalescence take place analogously to the case of cylindrical pillars and the pillar substrate gets filled with oil in an approximately square-like pattern[31]. Different from the cylindrical pillars (Figure 2 and Figure 3); here each pillar accommodates several droplets due to the large pillar size. Moreover, droplets also attach to the side walls and the pillars backsides, Figure S15. The side facing the flow direction is not preferred over the side turned away from the flow direction regarding more frequent drop attachment. Figure S18c compares the filling kinetics observed with cylindrical pillars with the filling kinetics for square micropillars. Change of oil or pillar geometry naturally leads to different filling velocities. The sharp steps in the time evolution of the coverage are caused by coalescence of oil patches outside the field of view. The coalesced oil domains spread onto the bottom substrate within the field of view.

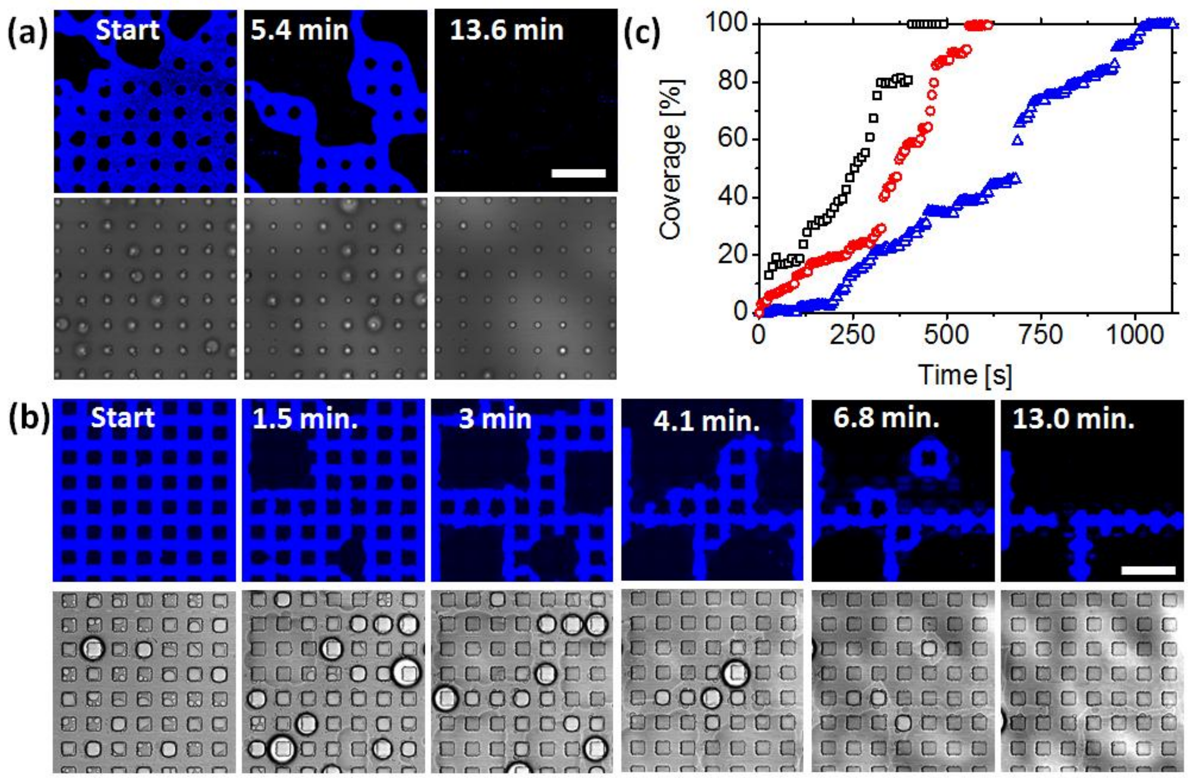

Figure S18. The generic nature of the filling and lubricant replenishment mechanism. a) Filling of an initially water-filled (blue = water) micropillar array (black) with the commercial lubricant (black) poly-α-olefin (PAO). The oil gradually replaces the water. Top row: fluorescence channel time evolution image sequence. Bottom row: transmission channel time evolution image sequence. Pillars' geometry: diameter = 5 µm, center-to-center spacing P = 20 µm, pillar height h = 10 µm. Scale bar: 50 µm. b) A substrate of square micropillars is filled with silicone oil. Top row: The fluorescence channel time evolution image sequence. Bottom row: The transmission channel time evolution image sequence. Pillars' geometry: width = 20 µm, center-to-center spacing P = 40 µm, pillar height h = 10 µm. Scale bar: 80 µm. The imaging plane lied 2 µm above the bottom surface of the micropillar array. c) Time evolution of the coverage for three different filling experiments with poly-α-olefin (red circles) and silicone oil with cylindrical (standard case, black squares) and square (blue triangles) micropillars. Apart from changing the oil and pillar geometry, the experimental conditions were identical. Flow velocity: 77 mm s^{-1}.

In the main text successful filling of the micropillar structure with silicone oil and PAO is demonstrated. The structure can also be successfully filled with Krytox® 103 and Fluorinert™ FC-70 as is shown in Figure S19 showing droplet attachment and growth to the micropillars. Successful filling is also observed in the case of customary food-grade olive oil (not shown).

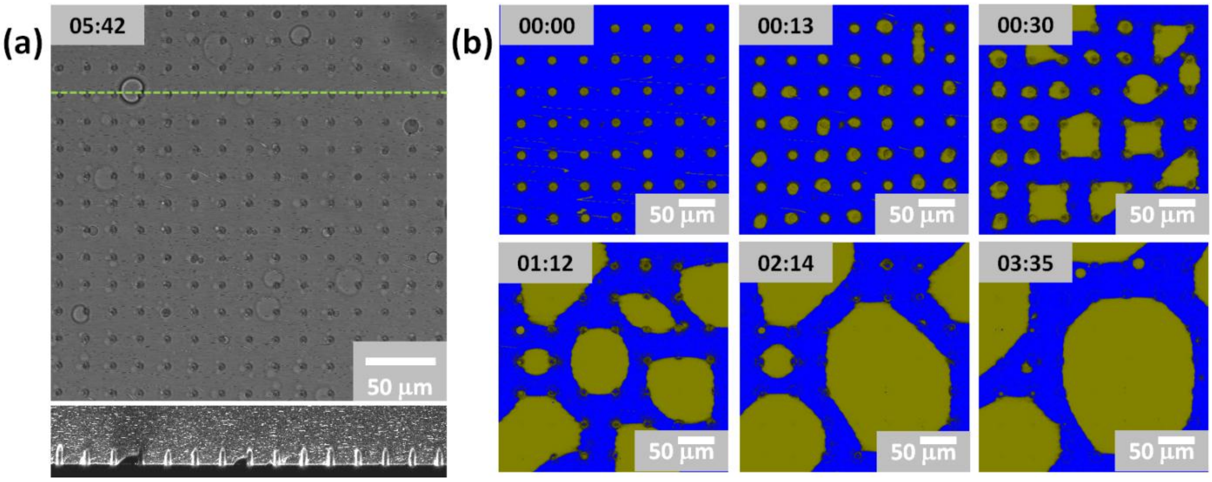

Figure S19. a) Filling of micropillar array with Krytox® 103. The filling mechanism works analogously to the case of using silicone oil or PAO as lubricating oil. The upper image shows a transmission channel top-view snapshot of the filling process at 5.42 min. after starting the filling experiment; the image on the bottom shows a transmission channel side view snapshot along the green line drawn above at the same time. b) Filling of a micropillar array with Fluorinert™ FC-70. The images display a time evolution of the filling process in the form of overlay images originating from the fluorescence channel (water: blue, oil: yellow).

For all the lubricants contained in the oil-and-water emulsions used for the filling experiments in this work (silicone oil, PAO, Krytox® 103, Fluorinert™ FC-70), the negative charge of the oil drops had to be compensated by adding 500 $\mu g L^{-1}$ of CTAB to the water phase before emulsification. In the main text, it is stated that a flow-induced separation of the less viscous component to the walls is unlikely to be the cause of the successful filling.

Below, in Figure S20, an experiment supporting this expectation is presented. In the standard filling experiments reported in this manuscript based on an oil-in-water emulsion containing 2 wt.-% 50 cSt silicone oil as well the positively charged surfactant CTAB at a concentration of 500 μgL^{-1}, the viscosity ratio of the lubricant to aqueous phase is $\eta_{oil}/\eta_{water} \approx 50$. To test whether the viscosity ratio matters, we conducted the same experiment using a ratio $\eta_{oil}/\eta_{water} \approx 50$, by exchanging pure water as the aqueous phase by a water-glycerol mixture having a viscosity of ≈ 100 mPa·s; a change by a factor of 100. Still, the filling mechanism works analogously, *i.e.* oil droplets (dyed yellow) attach to pillars, growth through coalescence, and descend onto the substrate. Water is replaced by oil at the pillars.

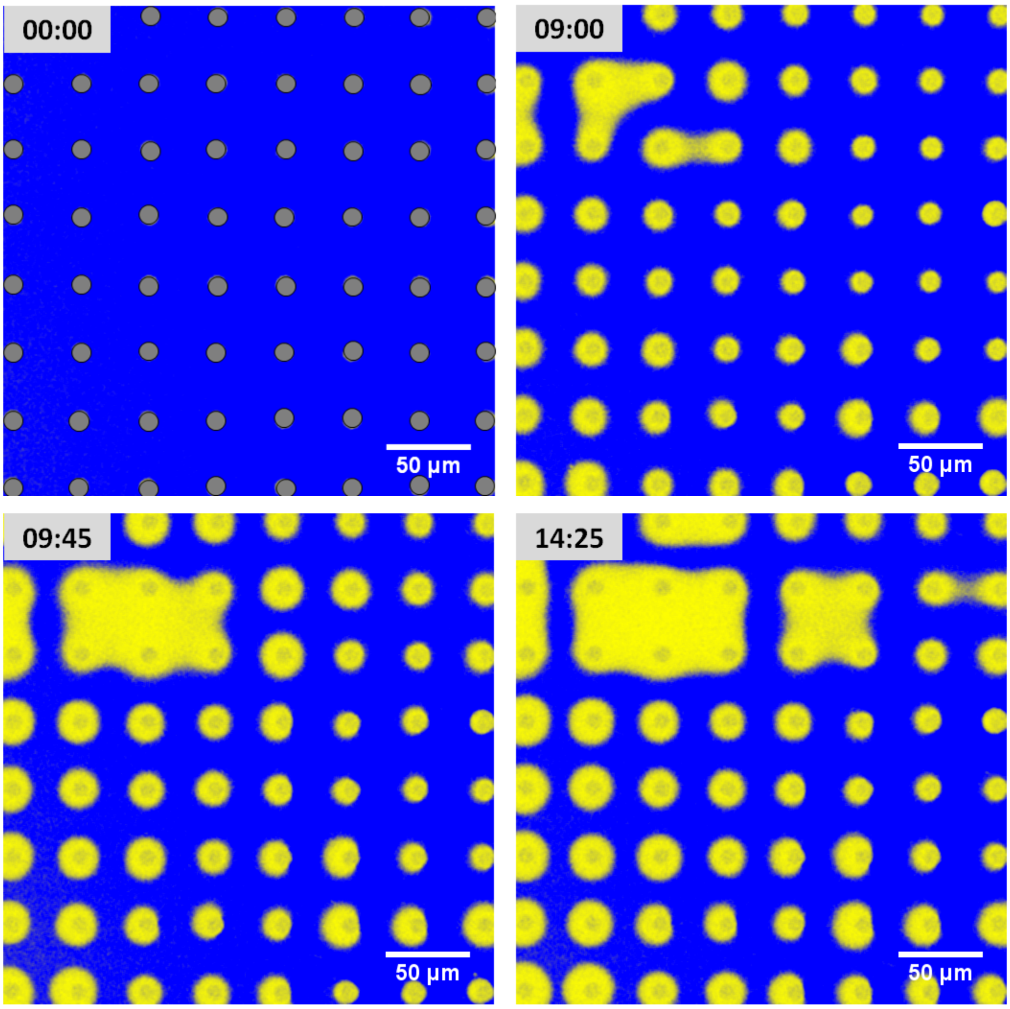

Figure S20. Filling experiment conducted with an emulsion whose continuous phase consisting of a water-glycerol mixture having a viscosity of 100 cSt. The concentration of CTAB was kept at 500 μgL^{-1}. The discontinuous/dispersed phase consists of 50 cSt silicone oil (PDMS), η_{oil}/η_{water} = 0.5. The filling takes place analogously to the standard case of oil-in-water emulsions, η_{oil}/η_{water} = 50, whose continuous phase is water. This supports that a flow-induced separation of the less viscous component to the walls of the micropillars or a certain viscosity ratio of the working fluid to the viscosity of the working fluid is unlikely to be the decisive factor for the successful filling of the structure.

S3.9 Generic Nature of Flow-Induced Lubricant-Replenishment from Emulsions

Lubricant-replenishment from the flow of emulsions works analogously for a broad range of lubricant-solid combinations and flow velocities. However, flow and positively charged surfactant are crucial. Firstly, the presence of flow is necessary to facilitate the attachment and growth of droplets, Figure S21. At some point, the flow in the filling experiment was stopped (indicated by time 00:00 in upper image) and droplets attached to pillar have remained unchanged for 15 minutes; they neither grow, nor detach, nor descend to the bottom substrate. It has to be noted that in the case of a lubricating oil less dense than water, buoyancy will keep the oil from contacting the bottom substrate as well as pillars. Secondly, without the addition of a surfactant, the underlying structure will not be filled with oil.

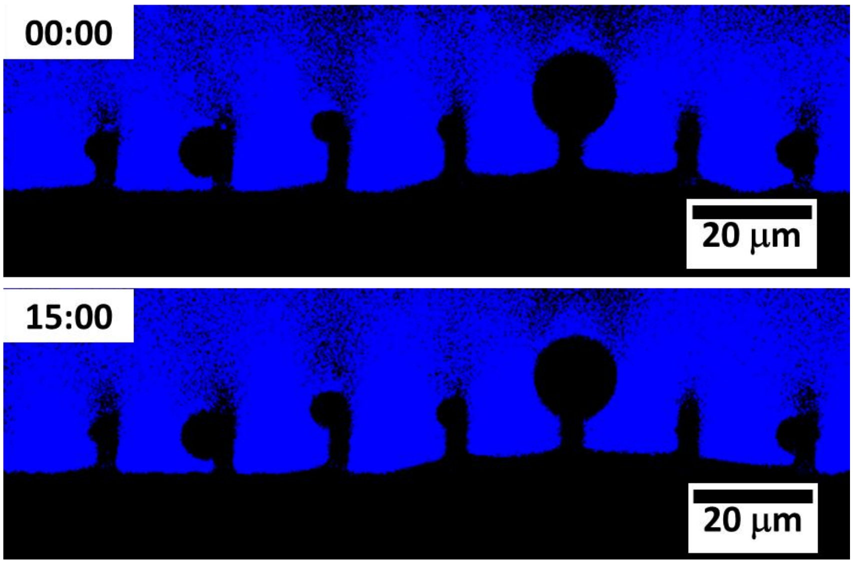

Figure S21. Influence of flow on the filling kinetics. After partially filling an OTS-coated micropillar array with silicone oil (contained in an oil-in-water emulsion, 2 wt.-% of silicone oil, 500 µg L^{-1} of CTAB) the flow was turned off. Even after 15 minutes, the filling did neither progress nor change.

For hydrophilic substrates, filling does not take place. The hydrophobic (oleophilic) nature of the underlying solid substrate needs to be preserved, as is shown in Figure S22, in order to facilitate filling.

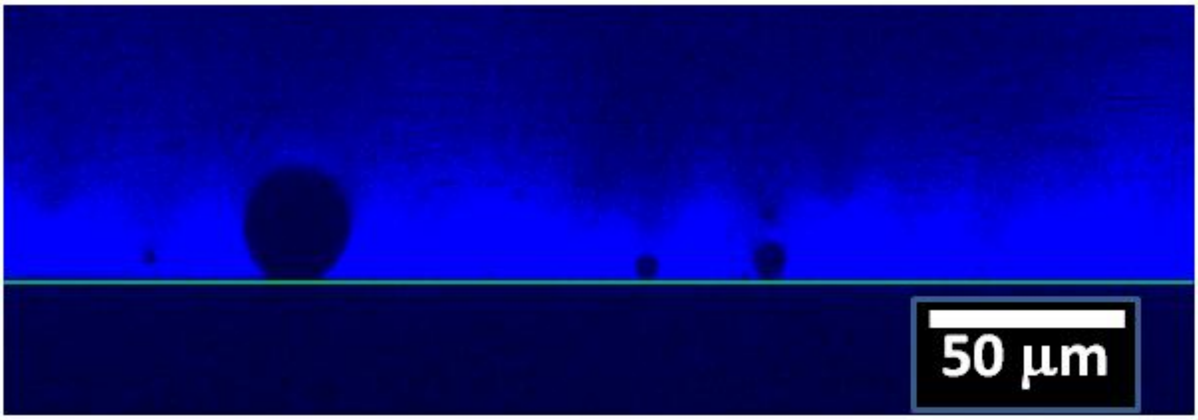

Figure S22. Side view fluorescence channel image (blue = water, black = oil droplets and bottom substrate, green line = boundary between solid substrate and emulsion) recorded in-

between pillars reveals that on a hydrophilic substrate consisting of oxygen-plasma-activated glass slides coated with SU-8 the oil droplets do not spread. Hence, no filling takes place.

When circulating the emulsion over the micropillar array, the number of drops adhering to the surface or merging with other drops or the oil film cannot be reliably predicted. However, we can estimate it from the filling rate and the amount of lubricant sheared off by the flow. The total volume of oil required to fill the structure completely is $6.1\cdot10^8$ µm^3. With a total filling time of 4 min the filling rate is thus $2.5\cdot10^6$ µm^3 s^{-1}. Assuming an average drop radius of 2 µm the filling rate is $7.6\cdot10^4$ drops s^{-1}. The amount of depleted lubricant, Equation 2 in the main text, in the flow cell is $6.6\cdot10^5$ µm^3 s^{-1} corresponding to $2\cdot10^4$ drops s^{-1}. At a flow rate of 7 ml min^{-1}, which corresponds to an average flow velocity = 77 mm s^{-1}, and an oil concentration of 2 wt.-% approximately $7\cdot10^7$ drops s^{-1} pass the flow channel. The flow of oil drops thus exceeds the amount of depleted lubricant by more than three orders of magnitude. Both the shear-induced depletion and the amount of oil droplets passing through the flow channel are equally dependent on the flow rate Q. Therefore, the ratio of upper limit of the filling rate $\dot{V}_{max}$ to the amount of depleted lubricant q_d does not change with the flow velocity, and always remains at a value of the order of 100, *i.e.* the upper limit of the filling rate $\dot{V}_{max}$ remains almost two orders of magnitude higher than the amount of depleted oil (Figure S23) q_d. Indeed, within the experimental range, the amount of circulated lubricant is sufficient to fill the structure independently on the flow velocity and even if the flow is reduced between the micropillar arrays.[32] Flow field considerations suggest that a minimum flow velocity is necessary to facilitate transport of oil droplets in-between the pillars and to the bottom substrate.[33]

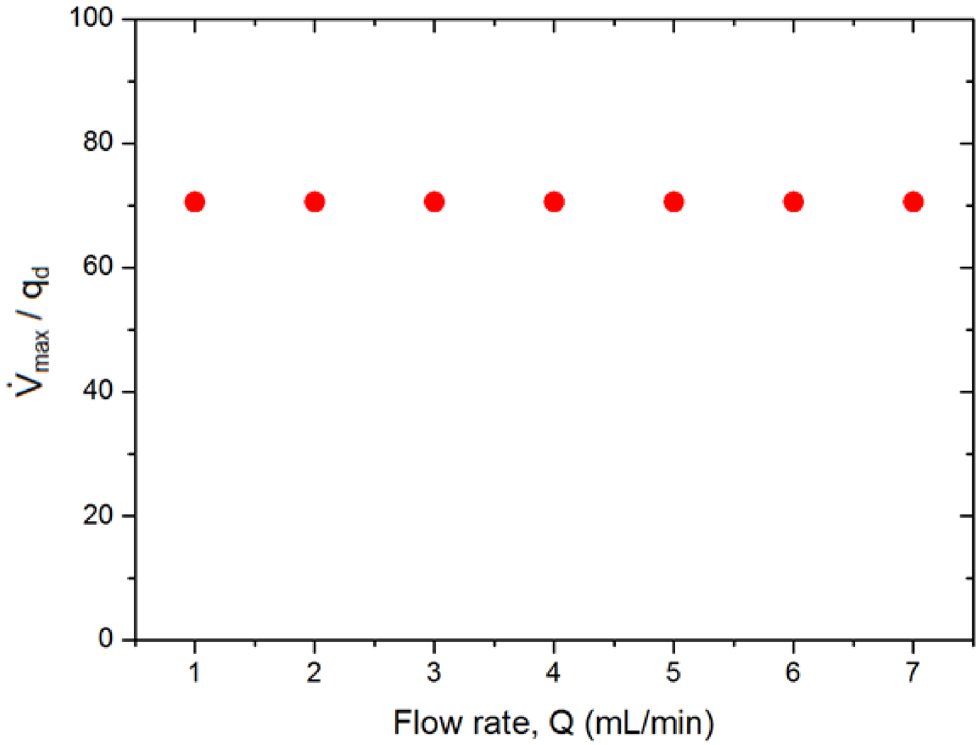

Figure S23. Ratio between the upper limit of the filling rate $\dot{V}_{max}$ to the amount of depleted lubricant q_d ($\dot{V}_{max}/q_d$) as a function of flow rate Q.

S3.10 Hydrodynamic Drag Force

Theoretically the hydrodynamic drag force of water (viscosity η_w = 1 mPa s) F_{hyd} should prevent coalescing and filling[11, 17]. For spheres $F_{hyd} = -\frac{6\pi\eta_w v R^2}{x}$ diverges if the distance x between the particle of radius R and the surface approaches zero. Because drops are deformable, no analytical expression exists. Still, only at sufficiently high velocity v or long contact times; the water film separating the drop and the pillar is sufficiently thinned during the impact that a defect can induce rupturing of the water film.[18]

Supplementary References

[25] P. Jungwirth, *Faraday Discuss*. **2009**, 141, 9.

[26] a) A. J. Babchin, L. L. Schramm, *Colloids Surf. B Biointerfaces* **2012**, 91, 137; b) Q. Li, X.-H. Wang, *RSC Adv.* **2017**, 7, 51426.

[27] a) Y. I. Frenkel, J. *Exptl. Theoret. Phys. (USSR)* **1948**, 18, 2; b) N. Gao, F. Geyer, D. Pilat, S. Wooh, D. Vollmer, H.-J. Butt, R. Berger, *Nat. Phys.* **2017**, 14, 191.

[28] E. B. Dussan V, *J. Fluid Mech*. **1987**, 174, 381.

[29] L. R. Rudnick, *Lubricant Additives*, CRC Press, Boca Raton **2017**.

[30] a) Wong, T. S., S. H. Kang, S. K. Tang, E. J. Smythe, B. D. Hatton, A. Grinthal, J. Aizenberg, *Nature* **2011**, 477, 443; b) P. S. Brown, B. J. Bhushan, *J. Colloid Interface Sci.* **2017**, 487, 437.

[31] C. Pirat, M. Sbragaglia, A. M., Peters, B. M., Borkent, R. G. H. Lammertink, M. Wessling, D. Lohse, *Europhys. Lett.* **2008**, 81, 66002.

[32] D. Schaffel, K. Koynov, D. Vollmer, H.-J. Butt, C. Schonecker, *Phys. Rev. Lett.* **2016**, 116, 134501.

[33] A. Osorio-Nesme, A. Delgado, *Fluid Dyn. Res*. **2017**, 49, 055502.
